# Exercise Intensity and Circulating Exerkine Responses: A Narrative Review of Selected Molecules

**DOI:** 10.3390/biom16060852

**Published:** 2026-06-10

**Authors:** Yanqi Zhao, Tutu Wang, Xinuan Zhang, Wange Wang, Yu Fu, Ismail Laher, Shunchang Li

**Affiliations:** 1 Sports Medicine Key Laboratory of Sichuan Province, Institute of Sports Medicine and Health, Chengdu Sport University, Chengdu 641418, China; 2 Department of Sport and Fitness Sciences, Gdansk University of Physical Education and Sport, 80-336 Gdansk, Poland; 3 Department of Pharmacology and Therapeutics, Faculty of Medicine, University of British Columbia, Vancouver, BC V6T 1Z3, Canada

**Keywords:** exerkines, exercise intensity, health benefit

## Abstract

Exerkines are bioactive molecules released in response to physical exercise and are considered important mediators of systemic adaptations. While previous research has largely focused on the effects of exercise modalities, the role of exercise intensity in regulating exerkine responses remains unclear. This narrative review summarizes findings on the effects of different exercise intensities on nine circulating exerkines with sufficient available data in healthy populations, including irisin, follistatin-like 1, myostatin, fibroblast growth factor 21, follistatin, leptin, adiponectin, apelin and brain-derived neurotrophic factor, without systematically covering all known exercise-responsive molecules. Given the narrative design of this review, the findings should be interpreted as descriptive and hypothesis-generating rather than as definitive evidence of intensity-dependent effects. The included studies show that acute exercise is associated with changes in several exerkines, with some direct within-study comparisons reporting larger responses under higher-intensity exercise conditions, whereas others exhibit increases, decreases, or no measurable changes across intensities. In contrast, studies examining chronic exercise interventions report changes in some studies and no measurable differences in others. Overall, the current evidence in this review suggests that exercise intensity may influence exerkine responses under some conditions, particularly during acute exercise, although the available findings remain limited and inconsistent across studies.

## 1. Introduction

Exercise is widely recognized as a central pillar of public health and wellbeing [1]. The World Health Organization (WHO) recognizes regular physical activity as a positive intervention in the prevention and control of chronic diseases worldwide, underscoring its important role in improving population health. Regular exercise not only enhances cardiopulmonary fitness, muscular strength, and weight regulation but also reduces the risk of cardiovascular and other chronic diseases [2]. In contrast, physical inactivity is a major risk factor for both reduced physical and mental health and contributes to the development of metabolic disorders that increase the risk of disability and premature mortality, creating substantial socioeconomic burdens on families and society [3]. This has led to the recommendation by the WHO that adults engage in 150–300 min of moderate-intensity or 75–150 min of vigorous-intensity physical activity per week, or a combination of both, to achieve optimal health benefits [4].

Exercise intensity influences the health benefits elicited by physical activity [5]. Exercise imposes physiological stress on the body, causing both acute responses and long-term adaptations. Distinct intensity domains are associated with differential metabolic, cardiovascular, and neuromuscular demands, thereby contributing to variability in physiological outcomes. Moderate-intensity exercise is commonly associated with improvements in cardiovascular function, metabolic regulation, and overall functional capacity, particularly when performed for longer durations [6,7]. Low-intensity exercise, although characterized by a lower acute physiological load, contributes to energy expenditure, improved recovery processes, and the maintenance of baseline physiological function, particularly in populations with limited exercise tolerance [8]. High-intensity exercise, by contrast, imposes greater metabolic and mechanical stress and is associated with pronounced adaptations in cardiorespiratory fitness, insulin sensitivity, and skeletal muscle function [9,10]. Training modalities characterized by higher intensities induce substantial physiological responses over a shorter time frame [11,12]. Collectively, these observations underscore the central role of exercise intensity in shaping the nature and magnitude of physiological adaptations to physical activity.

**Figure 1:**
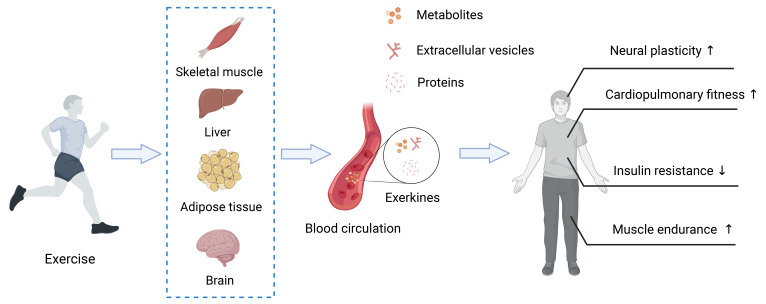
Effects of exercise-derived exerkines from different tissues on body variables. Physical exercise stimulates multiple tissues, including skeletal muscle, liver, adipose tissue, and brain, to release an array of diverse bioactive molecules collectively termed exerkines. These factors—comprising proteins, metabolites, and extracellular vesicles—are secreted into the circulation and act through endocrine, paracrine, and autocrine pathways to mediate inter-organ communication. Circulating exerkines exert systemic effects by regulating a wide range of physiological processes, including, but not limited to, neural plasticity, cardiopulmonary fitness, muscle function, and metabolic homeostasis. These coordinated responses collectively contribute to the broad health benefits associated with regular physical activity.

The concept of exerkines provides a novel theoretical framework for understanding the systemic health benefits induced by exercise. The term “exerkines” was first introduced by Adeel Safdar in 2016 to describe a group of biologically active factors that are released into the circulation in response to physical activity and that act as key mediators of exercise-induced systemic adaptations [13]. These exercise-induced signaling molecules facilitate communication between tissues and organs, thereby contributing to the integration of physiological responses to exercise at the whole-body level. Importantly, exerkines comprise a heterogeneous group of molecular species, including peptides, metabolites, and nucleic acids such as DNA, mRNA, and microRNAs, reflecting their diverse origins and mechanisms of action [13]. They exert their effects through autocrine, paracrine, and endocrine mechanisms, enabling coordinated regulation across multiple systems. Although initially associated with skeletal muscle-derived factors, subsequent research has broadened the concept of exerkines to include exercise-related circulating factors released from multiple tissues, including skeletal muscle, adipose tissue, the liver, and the brain. These factors mediate inter-organ communication and contribute to the integration of physiological responses to exercise at the whole-body level [14]. Numerous exerkines have been identified and reported to exhibit diverse biological functions, including the regulation of glucose and lipid metabolism, anti-inflammatory and antioxidant properties, and the promotion of neuroplasticity [14] (Figure 1).

Among the many factors influencing the expression of exerkines, exercise intensity is considered a key variable [5]. However, a clear consensus has yet to emerge regarding how exercise intensity influences exerkine responses. This review examines how different exercise intensities influence circulating levels of exerkines derived from various tissues in healthy populations, and considers evidence from both acute single-bout exercise studies and chronic exercise training interventions. Given the heterogeneity of exerkines and the variability and limited number of available studies for individual molecules, this review focuses on a subset of exerkines based on the availability of published studies reporting exercise-induced responses, particularly when information related to exercise intensity was reported. This approach reflects the current state of the literature and is intended to enable a focused discussion rather than provide a comprehensive overview of all known exerkines.

This review focuses on characterizing how circulating exerkine levels change in response to different exercise intensities and is not meant to provide a comprehensive evaluation of the health effects of exerkines or their mechanistic roles in exercise adaptation. Accordingly, it aims to identify common patterns and variability in exerkine responses across different exercise intensities and to summarize current evidence regarding potential associations between exercise intensity and circulating exerkine responses, thereby providing a theoretical foundation for the scientific development of exercise prescriptions.

## 2. Materials and Methods

This work was designed as a narrative review rather than a systematic review or meta-analysis and does not claim to be systematic or exhaustive; therefore, no formal PRISMA flow diagram, risk of bias assessment, or quantitative meta-analysis was performed. Relevant literature was identified through searches of PubMed and Google Scholar from database inception to March 2026, supplemented by manual screening of reference lists from relevant reviews and original studies. A literature screening flowchart is provided in Supplementary Figure S1. The selection of specific exerkines included in this review was guided by the availability of published studies reporting exercise-induced responses, particularly those providing information on exercise intensity. Representative examples of exercise-responsive molecules not included in the present review and the reasons for non-inclusion are provided in Supplementary Table S1. Search terms included combinations of “exerkines,” “exercise intensity,” “irisin,” “Fstl1,” “follistatin-like protein 1,” “MSTN,” “myostatin,” “FGF21,” “fibroblast growth factor 21,” “FST,” “follistatin,” “leptin,” “adiponectin,” “apelin,” “brain-derived neurotrophic factor,” “BDNF,” “high-intensity exercise,” “moderate-intensity exercise,” “low-intensity exercise,” “resistance training,” and “endurance exercise.” Detailed literature search strategies are provided in Supplementary Table S2.

Studies were considered eligible if they were conducted in humans, included healthy participants, reported circulating exerkine responses before and after exercise, and provided information on exercise intensity. Animal studies, disease-specific intervention studies, and studies without clearly defined exercise intensity or circulating exerkine outcomes were not considered for the main synthesis. For each included study, information on publication year, participant sex and age, sample size, exercise type, exercise intensity, exercise frequency and duration, time of blood sample and circulating exerkine responses was extracted and summarized in Table 1, Table 2, Table 3 and Table 4.

The reporting of exerkine concentration changes varied substantially across studies. Some studies presented absolute concentrations with specific units (e.g., ng/mL, pg/mL), while others reported only relative or percentage changes without providing raw concentration values. To improve comparability across studies, we primarily used percentage change to quantify exercise-induced alterations in circulating exerkine levels. These percentage changes were calculated relative to baseline (pre-exercise) values. When absolute values were available, percentage changes were calculated where appropriate; when only percentage changes were reported, these were directly extracted. For studies reporting multiple post-exercise time points, the time point showing the peak change in exerkine concentration was selected for presentation, and the corresponding sampling time and reported value were indicated. Baseline and post-exercise values (means ± SD), when available, are provided in Supplementary Tables S5–S13. Consistent with the percentage change data presented in the main tables, for studies reporting multiple post-exercise time points, the values corresponding to the peak exerkine response were selected.

## 3. Exercise Intensity as a Modulator of Health Benefits

### 3.1. Overview and Classification of Exercise Intensity

Exercise intensity is a key indicator for assessing exercise load, representing the magnitude of physical exertion per unit of time and the corresponding physiological stimulation of the body. It is typically classified into two categories: absolute intensity and relative intensity [15]. Absolute intensity refers to the quantifiable external physical load experienced by the body during exercise, which is an objective constant independent of the individual’s physiological or functional status. For example, running at a fixed speed (e.g., 10 km/h) or cycling at a fixed power output (e.g., 150 W) represents absolute intensity. Relative intensity, on the other hand, reflects the physiological stress level the body experiences in response to exercise load, taking into account individual differences in physiological function and capacity. For instance, exercising at 70% of maximum heart rate (HRmax) or 60% of maximal oxygen uptake (VO_2_max) represents relative intensity and is more suitable for individualized exercise prescriptions. This intensity is often assessed using individualized physiological parameters, including subjective fatigue (rating of perceived exertion, RPE), VO_2_max, anaerobic threshold (AT), HRmax, heart rate reserve (HRR), metabolic equivalents (METs), and one-repetition maximum (1RM), among others.

This review uses the classification standards outlined by the American Heart Association (AHA) for physical activity assessment [16]: (a) high-intensity exercise is defined as VO_2_max (%) ≥ 60, HRmax (%) ≥ 70, HRR (%) ≥ 60, or METs ≥ 6; (b) moderate-intensity exercise is defined as VO_2_max (%) 45–59, HRmax (%) 50–69, HRR (%) 45–59, or METs 3.0–5.9; and (c) low-intensity exercise is defined as VO_2_max (%) ≤ 44, HRmax (%) ≤ 49, HRR (%) ≤ 44, or METs ≤ 2.9. For resistance training, the intensity is typically measured as a percentage of one-repetition maximum (1RM) [17]. Low intensity is generally defined as 60% < 1RM, moderate intensity as 60–80% 1RM, and high intensity as ≥80% 1RM. While these thresholds are widely used, variations in intensity definitions across studies should be considered when interpreting and comparing findings.

### 3.2. Role of Exercise Intensity in Providing Health Benefits

Exercise intensity is an important determinant of the health benefits of physical activity by regulating the degree and nature of physiological perturbations induced [18]. Different intensity domains induce distinct metabolic and mechanical demands, which in turn influence the internal environment of the body and the signals that drive subsequent adaptive processes. At lower to moderate intensities, exercise is typically performed under relatively stable physiological conditions, with energy demands primarily supported by aerobic metabolism [19]. This results in sustained but moderate alterations in substrate utilization, oxygen consumption, and hemodynamics, which contribute to improvements in metabolic efficiency, cardiovascular function, and fatigue resistance [20]. In contrast, higher-intensity exercise leads to more pronounced but transient disturbances in homeostasis, characterized by rapid energy turnover, increased reliance on anaerobic metabolism through phosphocreatine breakdown and glycolysis, accumulation of metabolites such as lactate, and greater mechanical stress on skeletal muscles [21,22]. These changes not only impose higher physiological demands but also generate stronger biochemical and mechanical signals that can trigger more rapid or pronounced adaptive responses [23,24]. These intensity-dependent differences in physiological perturbation provide a basis for variability in downstream biological processes, including the release and regulation of exercise-induced circulating factors [5]. Therefore, exercise intensity should be considered as a key regulator of the internal conditions that shape both systemic adaptations and molecular responses to physical activity.

## 4. How Exercise Intensity Modulates Exerkine Responses

### 4.1. Overview of Exerkines

Exerkines are broadly defined as exercise-responsive signaling molecules released from multiple organs, including skeletal muscle, liver, adipose tissue, brain, and other metabolically active tissues, in response to acute and/or chronic physical activity [13]. These factors encompass a wide spectrum of bioactive substances, such as peptides, metabolites, nucleic acids, and extracellular vesicles, and exert their effects through endocrine, paracrine, and autocrine pathways, thereby coordinating systemic physiological adaptations to exercise [25] (Figure 1). Since the identification of interleukin-6 (IL-6) as the first myokine in 2000 [26], numerous other exercise-responsive factors have been discovered in the nervous system, adipose tissue, skeletal muscle, and liver [14].

The term “exerkines” is conceptually distinct from traditional organ-based classifications such as myokines, adipokines, hepatokines, and neurotrophic factors. While these terms are defined strictly according to their tissue of origin, exerkines are defined based on their responsiveness to exercise stimuli [13], regardless of their source. Therefore, exerkines represent a functional category that may encompass multiple classes of exercise-responsive molecules derived from different organs and tissues [27].

Based on their primary tissue of origin or discovery, exerkines can be broadly categorized into the following groups [14]: myokines, such as irisin, myostatin (MSTN), and follistatin-like protein 1 (Fstl1), which are secreted by skeletal muscle cells; hepatokines, such as fibroblast growth factor 21 (FGF21) and follistatin (FST), which are secreted by the liver; adipokines, such as leptin, adiponectin, and apelin, which are secreted by adipocytes; neurotrophic factors, with brain-derived neurotrophic factor (BDNF) as a typical example (Figure 1). This classification reflects a practical framework for organization rather than strict biological boundaries, as many exerkines may originate from multiple tissues and exert pleiotropic effects. These exerkines exert pleiotropic effects via multiple targets and pathways by orchestrating energy metabolism, oxidative stress regulation, tissue repair, immune homeostasis, neuroplasticity, and cardiovascular function. Collectively, these changes encompass the benefits of exercise at multiple levels, including health promotion, wellbeing and disease prevention [14] (Figure 1).

Exerkines participate in systemic regulation through multi-target and multi-pathway mechanisms: at the molecular level, they coordinate the neuro–immune–endocrine network, influencing lipid metabolism, insulin sensitivity, mitochondrial function, inflammatory responses, and neuroplasticity [28]. In disease prevention and management, exerkines are potential therapeutic targets for obesity, type 2 diabetes, cardiovascular diseases, neurodegenerative disorders, and sarcopenia [25,29]. In the context of health promotion, they contribute to maintaining energy balance, enhancing exercise capacity, facilitating recovery, and improving psychological well-being, thereby optimizing physiological function and quality of life [30]. Collectively, these findings provide a theoretical foundation for developing targeted health interventions based on exerkine-mediated mechanisms.

Exercise intensity is considered an important factor that may influence exerkine expression [5]. Different exercise intensities may influence exerkine levels through distinct metabolic and mechanical stress pathways, with some studies suggesting that higher-intensity exercise is associated with larger responses in some exerkines [31]. However, evidence from other studies suggests that this relationship is not uniform for all exerkines, with variability observed depending on the type of molecule, exercise conditions, and population characteristics [32]. These findings suggest that exerkine responses may vary across different exercise intensity conditions. Therefore, it remains necessary to systematically examine how different exerkines respond to varying exercise intensities.

### 4.2. Exercise Intensity Modulates Myokine Responses

Skeletal muscle is an important source of exercise-responsive myokines [14]. In this section, we summarize how selected skeletal muscle-derived exerkines, such as irisin, Fstl1, and MSTN, respond to different exercise intensities.

#### 4.2.1. Irisin

Irisin is an exercise-induced myokine, primarily generated through the peroxisome proliferator-activated receptor gamma coactivator-1α (PGC-1α)/fibronectin type III domain-containing protein 5 (FNDC5) signaling pathway in skeletal muscle [33,34]. It promotes energy expenditure by activating brown adipose tissue and facilitating the browning of white adipose tissue, thereby contributing to body weight control and improved metabolic health [35,36]. In addition, irisin improves insulin sensitivity, regulates glucose metabolism, reduces inflammation, and exerts protective effects on the cardiovascular and nervous systems [33,37]. It also positively influences exercise capacity, maintains musculoskeletal homeostasis via mitochondrial quality control mechanisms, and regulates muscle growth, regeneration, satellite cell activation, and metabolic activity [38,39]. Given its key role in exercise-induced adaptations, irisin has been extensively investigated for its responsiveness to exercise intensity.

Studies on the effects of acute exercise suggest that irisin levels may respond rapidly to acute exercise. Some studies included direct within-study comparisons of different exercise intensities. A study by Colpitts et al. reported that acute high-intensity interval training (HIIT) elevated circulating irisin concentrations during exercise in adolescents, with increases observed at multiple time points (7–28 min) and a peak increase of up to 81%, whereas moderate-intensity continuous exercise (MICE) induced no significant changes [31]. Similarly, Tsai et al. demonstrated that a single session of HIIT increased irisin levels (~8.4%) in middle-aged and older adults, while MICE caused no measurable changes [40]. Likewise, Tsuchiya et al. reported that high-intensity endurance exercise (80% VO_2_max) induced sustained elevations in irisin at 6 and 19 h post-exercise (18% and 23%, respectively), whereas low-intensity exercise (40% VO_2_max) reduced irisin levels, with concentrations decreasing by approximately 38% immediately after exercise [41]. A study of trained adolescent swimmers by Huh et al. further showed that HIIT elicited a rapid (~30%) and transient increase in irisin immediately after exercise, while MICE induced only non-significant fluctuations [42]. Collectively, these direct within-study comparisons suggest that different exercise intensities may be associated with different acute irisin response patterns under some conditions. In addition to the direct comparison studies, single-intensity studies have also reported exercise-induced changes in irisin levels. For example, acute high-intensity resistance training induced transient increases (~23%) in irisin, peaking within the early recovery phase [43] (Table 1).

In the context of this review, evidence from chronic exercise is limited. The available chronic studies primarily consist of single-intensity interventions rather than direct within-study intensity comparisons. Adilakshmi et al. reported substantial elevations (~248%) after 8 weeks of high-intensity resistance training [44], whereas long-term moderate-intensity resistance training did not significantly change irisin concentrations [45] (Table 1). Based on the limited number of studies included, these findings suggest that long-term adaptations of irisin to exercise may be influenced by exercise intensity; however, this observation should be interpreted with caution.

In summary, based on the studies included in this review, irisin levels respond to acute exercise, and some direct within-study comparisons reported larger irisin responses under higher-intensity exercise conditions, although variability across study conditions indicates that this relationship is not uniform. Evidence from chronic exercise interventions included in this review is limited. Overall, the available evidence in this review suggests that exercise intensity may influence irisin responses under some conditions, particularly during acute exercise.

#### 4.2.2. Follistatin-like 1 (Fstl1)

Fstl1 is a glycoprotein-type exerkine secreted primarily by skeletal muscle and the heart, among other tissues [46]. It plays critical roles in metabolic regulation, cardiovascular protection, and immune modulation by promoting lipolysis, inhibiting adipogenesis, and enhancing lipid mobilization, thereby improving insulin sensitivity and glucose–lipid metabolism [47,48]. In addition, the cardiovascular effects of Fst11 include suppression of cardiomyocyte apoptosis, stimulation of angiogenesis and cardiac repair, and reduction of myocardial fibrosis [49,50]. The anti-inflammatory effects of Fst11 occur by reducing pro-inflammatory cytokine levels and maintaining immune homeostasis [51].

Acute exercise studies indicate that circulating Fstl1 levels may increase following exercise, although the extent of this response varies across studies. The available evidence includes both direct within-study intensity comparisons and single-intensity exercise studies. For example, Görgens et al. reported that endurance exercise at 70% VO_2_max increased serum Fstl1 levels by approximately 29% at 30 min post-exercise [52]. Nam et al. observed an increase in circulating Fstl1 levels during exercise at 70% HRmax, with peak concentrations immediately post-exercise, reaching approximately 71% above baseline [47]. Sprint interval training elicited a rapid increase (~73%) immediately after exercise [53]. Notably, Ji et al. directly compared multiple exercise intensities within the same study and demonstrated that both HIIT and vigorous continuous exercise induced large increases in Fstl1 levels (~175% and 100%, respectively), whereas MICE did not produce significant changes [54]. In contrast, evidence from other studies suggests that moderate-intensity exercise may also elevate Fstl1 levels. For instance, Xu et al. reported a modest increase (~19%) following exercise [55] (Table 1). However, because this finding was derived from a separate study with different participant characteristics, exercise protocols, and sampling conditions, caution is warranted when comparing these responses with findings reported under higher-intensity exercise conditions in other studies.

In summary, based on the studies included in this review, Fstl1 appears to exhibit acute and transient increases following exercise. Direct within-study comparisons suggest that different exercise intensities may be associated with different Fstl1 response patterns under some conditions; however, the number of such studies remains limited. Therefore, the available evidence suggests that acute Fstl1 responses may be influenced by exercise intensity in some cases. Given the limited number of studies and the heterogeneity in study designs, these findings should be interpreted with caution. Notably, no chronic exercise intervention studies on Fstl1 were identified among the studies included in this review.

#### 4.2.3. Myostatin (MSTN)

MSTN, a member of the transforming growth factor-β (TGF-β) superfamily, is predominantly expressed and secreted by skeletal muscle and acts as a negative regulator of muscle growth and differentiation [56]. MSTN suppresses myocyte growth and regeneration by inhibiting satellite cell proliferation and differentiation, thereby limiting skeletal muscle mass [57,58]. In addition to its role in muscle biology, MSTN is also closely involved in metabolic regulation, as elevated MSTN expression is associated with obesity, insulin resistance, and type 2 diabetes, whereas its inhibition attenuates the progression of these conditions [59,60]. Therefore, modulation of MSTN not only influences exercise-induced muscle adaptation but also represents a potential therapeutic target for metabolic disorders.

Acute exercise studies suggest that circulating MSTN levels may decrease following exercise; however, the number of studies included in this review is limited. Willoughby et al. directly compared different resistance training intensities within the same study and observed a greater decrease (~35.7%) after high-load resistance training compared to lower-load conditions (~17%) [61]. In addition to direct comparison studies, single-intensity studies have also reported reductions in MSTN levels following acute exercise. For example, low-intensity resistance training reduced circulating MSTN levels, although the magnitude of change appeared smaller (~3.5%) [62] (Table 1).

Chronic exercise interventions included in this review also report reductions in circulating MSTN levels across different training protocols. Ziyaiyan et al. reported a decrease (~19%) following 8 weeks of high-intensity circuit training (HICT) [63]. In addition, Shabkhiz et al. observed a reduction (~17.1%) after 12 weeks of moderate-intensity resistance training [64], and Arazi et al. reported a decrease (~23.2%) following 8 weeks of moderate-intensity exercise in postmenopausal women [65]. Furthermore, one study directly compared different exercise intensities within the same population and reported that moderate-intensity endurance training was associated with reductions (~16%) in MSTN levels, whereas HIIT did not result in significant changes within the same population [32] (Table 1). This inconsistency suggests that factors other than exercise intensity may influence MSTN responses. Since different exercise modalities were used across conditions, exercise modality may have acted as a potential confounder when interpreting the effects of exercise intensity.

Based on the studies included in this review, both acute and chronic exercise have been associated with reductions in circulating MSTN levels under some conditions. Direct within-study comparisons suggest that exercise intensity may influence MSTN responses, although the observed patterns differ across exercise conditions. In acute resistance exercise, higher-load conditions were associated with greater reductions in MSTN levels compared with lower-load conditions. In contrast, a chronic exercise study reported reductions following moderate-intensity endurance training, whereas HIIT did not produce significant changes within the same population. In addition, findings from single-intensity studies indicate that reductions in circulating MSTN levels have been observed across multiple exercise conditions.

**Table 1:** Effects of exercise intensity on myokine circulating levels.

Exerkine	Publication Year	Study Population	Exercise Type	Exercise Intensity	Frequency and Duration	Time of Blood Sample	Changes in Concentration	Reference
**Acute exercise**								
Irisin	2022	7 males and 7 females; Mean age: 17.14 ± 1.66 years; Baseline fitness levels: untrained	HIIT; MICE	MICE: (50% HRR); HIIT: (85–90% HRR)	MICE: Single session, 35 min; HIIT: Single session, 35 min; (total session, including rest intervals)	Baseline and during the training at 7, 14, 21, 28 min and immediately post-exercise.	MICE: no significant change; HIIT: ~81.0% increase (during the training)	[31]
	2021	10 males and 11 females; Mean age: 60.62 ± 4.96 years; Baseline fitness levels: untrained	HIIT; MICE	MICE: (50–55% HRR); HIIT: (70–75% HRR)	MICE: Single session; 30 min HIIT: Single session; 30 min (total session, including rest intervals)	Baseline and post-exercise.	MICE: no significant change; HIIT: ~8.4% increase (post-exercise)	[40]
	2014	6 males; Mean age: 22.5 ± 1.1 years; Baseline fitness levels: untrained	HICE; LICE	HICE: (80% VO_2_max); LICE: (40% VO_2_max)	HICE: Single session, 40 min; LICE: Single session, 20 min	Baseline and immediately after exercise, at 3, 6, and 19 h post-exercise.	HICE: ~18% increase 6 h and ~23% increase 19 h post-exercise; LICE: ~38% decrease (immediately post-exercise)	[41]
	2014	15 males; Mean age: 15.4 ± 0.2 years; and 15 females, mean age: 15.4 ± 0.3 years; Baseline fitness levels: trained	HIIT; MICE	MICE: (-); HIIT: (-)	MICE: Single session, 27 min 37 ± 22 s; HIIT: Single session, 32.23 ± 0.47 s	Baseline, immediately (in less than 5 min), 1 h, and 24 h post-exercise.	MICE: no significant change; HIIT: ~30% increase (immediately post-exercise)	[42]
	2015	7 males and 2 females; Mean age: 32 ± 9 years; Baseline fitness levels: trained	RT	High: (-)	Single session; 60 min	15 min prior to exercise, immediately post-exercise, and thereafter 1, 2, 4, 6 and 24 h post-exercise.	~23% increase (at 1 h post-exercise)	[43]
Fstl1	2013	8 males; Mean age: (-); Baseline fitness levels: trained	HICE	High: (70% VO_2_max)	Single session; 60 min	Baseline and immediately after the exercise session as well as 30 min and 120 min post-exercise.	~29% increase (at 30 min post-exercise)	[52]
	2024	29 males; Mean age: 24.28 ± 3.53 years; Baseline fitness levels: untrained	HICE	High: (70% HRmax)	Single session; 60 min	Baseline and immediately after the exercise session as well as 30 min, 60 min and 120 min post-exercise.	~71% increase (immediately after exercise)	[47]
	2020	10 males and 11 females; Mean age: 25.2 ± 1.8 years; Baseline fitness levels: untrained	MICE	Moderate: (60% VO_2_max)	Single session; 45 min	Baseline and 45, 60, and 120 min following the 45 min bout of exercise.	~19% increase (immediately after exercise)	[55]
	2021	8 males; Mean age: 20.3 ± 0.6 years; Baseline fitness levels: untrained	SIT	High: (maximal efforts)	Single session; Four bouts of 30 s all-out sprints	Baseline and immediately after the exercise session as well as 15, 30 and 120 min post-exercise.	~73% increase (immediately after exercise)	[53]
	2024	9 males; Mean age: 24.0 ± 0.4 years; Baseline fitness levels: untrained	HIIT; VICE; MICE	MICE: (55% HRR); VICE: (85% HRR); HIIT: (all out)	MICE, VICE: Single session; 30 min; HIIT: four bouts of 30 s exercise with 4 min rest intervals	Baseline and immediately after the exercise session as well as 30, 90 min post-exercise.	HIIT: ~175% increase (immediately after exercise); VICE: ~150% increase (at 30 min post-exercise); MICE: no significant change	[54]
MSTN	2022	10 males; Mean age: 23.2 ± 4.68 years; Baseline fitness levels: trained	RT	High: (80% 1RM); Low: (50% 1RM)	Single session; To volitional failure	Baseline and at 3 and 24 h post-exercise.	Low: ~17% decrease (at 24 h post-exercise); High: ~35.7% decrease (at 24 h post-exercise)	[61]
	2016	12 males; Mean age: 22.1 ± 2.1 years; Baseline fitness levels: trained	RT	Low: (55% 1RM)	15 repetitions per set; 3 sets	Baseline and 24 h post-exercise.	~3.5% decrease (at 24 h post-exercise)	[62]
**Chronic exercise**								
Irisin	2023	50 males; aged 21 to 35 years. Baseline fitness levels: untrained	RT	High: (70% HRmax)	Five times per week; 8 weeks	Baseline and 30 min after the last intervention.	~248% increase	[44]
	2014	17 males and 20 females; Mean age: 47 ± 7 years; Baseline fitness levels: untrained	RT	Moderate: (64–71% 1RM)	Three days per week; 24 weeks	After a 10 min supine resting period.	No change	[45]
MSTN	2023	9 males; Mean age: 15.17 ± 0.37 years; Baseline fitness levels: trained	HICT	High: (80–85% HRmax)	Three times per week; 8 weeks	48 h before training and 48 h after the last training session.	~19% decrease	[63]
	2021	9 males and 23 females; Mean age: 61 ± 12 years; Baseline fitness levels: untrained	MICE; HIIT	MICE: (60–70% HRmax); HIIT: (90% HRmax)	MICE: Three times per week, 12 week; HIIT: Three times per week, 2 week	Baseline and 24 h after completing the training program.	MICE: ~16% decrease; HIIT: no significant change	[32]
	2021	12 males, aged 65 to 78 years; Baseline fitness levels: untrained	RT	Moderate: (70% 1RM)	Three times per week; 12 weeks	One week before the training and 48 h after the last training.	~17.1% decrease	[64]
	2020	12 females; Mean age: 23.58 ± 3.84 years; Baseline fitness levels: untrained	RT	Moderate: (65–80% 1RM)	Three times per week; 8 weeks	48 h before training and 48 h after the last training.	~23.2% decrease	[65]

Abbreviations: HICE: High-Intensity Continuous Exercise; HICT: High-Intensity Circuit Training; HIIT: High-Intensity Interval Training; HRmax: Maximun Heart Rate; HRR: Heart Rate Reserve; LICE: Low-Intensity Continuous Exercise; MICE: Moderate-Intensity Continuous Exercise; RM: Repetition Maximum; RT: Resistance Training; SIT: Sprint Interval Training; VICE: Vigorous-Intensity Continuous Exercise; VO_2_max: Maximal Oxygen Uptake; “(-)” indicates that the specific numerical threshold for exercise intensity was not reported in the original study; “~” indicates approximate percentage changes reported in the original studies.

### 4.3. Exercise Intensity Modulates Hepatokines Responses

The liver, as a central metabolic organ, is a major source of circulating proteins and exercise-responsive cytokines such as FGF21 and FST. These exerkines influence glucose and lipid metabolism, promote the browning of white adipose tissue, and maintain cellular homeostasis, thereby playing key roles in systemic energy metabolism and related physiological processes [14].

#### 4.3.1. Fibroblast Growth Factor 21 (FGF21)

FGF21 is a 208-amino acid signaling protein belonging to the fibroblast growth factor family and is primarily secreted by the liver [66]. It plays a pivotal role in energy metabolism, particularly in the regulation of glucose–lipid homeostasis and insulin sensitivity [67]. FGF21 enhances glucose uptake and utilization, promotes fatty acid oxidation, and inhibits lipogenesis, thereby improving glycemic and lipid control [68]. In addition, it is involved in metabolic stress adaptation, such as during fasting, by stimulating thermogenesis in brown adipose tissue and regulating energy balance [69]. FGF21 also exerts anti-inflammatory, antioxidant, and cardioprotective effects [70].

Acute exercise studies suggest that circulating FGF21 levels may increase following exercise; however, findings vary across studies and conditions. Some studies included direct within-study comparisons of different exercise intensities. For example, Ji et al. demonstrated that both HIIT and vigorous continuous exercise increased FGF21 concentrations, whereas moderate-intensity exercise did not induce significant changes [54]. He et al. reported that both moderate- and high-intensity endurance exercise increased circulating FGF21 levels in the same participants, with greater elevations observed at higher intensity [71]. Similar findings were reported by Willis et al., who observed increases in FGF21 levels at both moderate and high intensities, with substantially greater increases during higher-intensity exercise [72]. Morville et al. reported that high-intensity endurance exercise significantly elevated FGF21, whereas high-intensity resistance training did not induce any changes in the same group of participants [73]. These direct within-study comparisons suggest that exercise intensity, and possibly exercise modality, may influence acute FGF21 responses under some conditions. In addition to the direct comparison studies, single-intensity studies have also reported exercise-induced increases in FGF21 levels. For example, moderate-intensity resistance training has been reported to increase circulating FGF21 levels, with peak concentrations observed approximately 24 h post-exercise [74] (Table 2).

Chronic exercise interventions included in this review are limited. The available chronic evidence consists primarily of single-intensity interventions rather than direct within-study intensity comparisons. One study reported that low-intensity training was associated with modest increases in circulating FGF21 levels over a longer-term intervention [75] (Table 2).

Based on the studies included in this review, increases in circulating FGF21 levels have been reported following exercise, although the magnitude and timing of responses vary across studies and conditions. Direct within-study comparisons from acute exercise studies suggest that different exercise intensities may be associated with different FGF21 response patterns under some conditions, with some studies reporting larger increases under higher-intensity exercise conditions. However, chronic exercise evidence remains limited and is primarily derived from single-intensity interventions. Given the limited number of studies, the available evidence should be interpreted with caution.

In the study by Morville et al. [73], both endurance exercise and resistance training were performed in the same participants under high-intensity conditions; however, only endurance exercise significantly increased circulating FGF21 levels, whereas RT did not induce measurable changes. This finding suggests that factors other than exercise intensity may contribute to variability in FGF21 responses. In particular, exercise modality may influence circulating FGF21 responses, as different exercise modes elicited distinct responses despite both being performed at high intensity.

#### 4.3.2. Follistatin (FST)

FST is a glycoprotein that primarily functions as an antagonist of TGF-β family members, particularly activins [76]. By binding to activins with high affinity, FST inhibits their receptor interactions, thereby regulating the hypothalamic–pituitary–gonadal axis and influencing reproductive function and follicular development [77]. Beyond the reproductive system, FST is expressed in multiple tissues, including skeletal muscle, liver, and adipose tissue, where it participates in cell proliferation, differentiation, and tissue repair [78,79]. In skeletal muscle, FST antagonizes myostatin, promoting muscle hypertrophy and growth, and is therefore closely associated with exercise adaptation and strength development [56].

Acute exercise studies suggest that circulating FST levels may increase following exercise; however, findings vary across studies and conditions. Some studies included direct within-study comparisons of different exercise intensities. For example, He et al. reported larger increases following high-intensity exercise (~259%) compared with moderate-intensity exercise (~141%) [71]. Willis et al. also observed higher peak values after high-intensity exercise, although increases occurred under both conditions [72]. However, Willoughby et al. reported that both low-load (50% 1RM) and high-load (80% 1RM) resistance training increased FST levels 24 h post-exercise, with a greater increase observed under low-load conditions [61]. These direct within-study comparisons suggest that exercise intensity may influence acute FST responses under some conditions, although the direction of the response is not uniform across studies. In addition to the direct comparison studies, single-intensity studies have also reported exercise-induced increases in FST levels. For example, moderate-intensity resistance training has been reported to increase circulating FST levels, with peak concentrations observed approximately 24 h post-exercise [74]. These findings suggest that increases in FST have been observed across different loading conditions within resistance training (Table 2).

Evidence from chronic exercise interventions is limited in the studies included in this review. The available chronic evidence consists primarily of single-intensity interventions rather than direct within-study intensity comparisons. One study reported that moderate-intensity resistance training was associated with increases in circulating FST levels (~49.1%) following a longer-term intervention [65] (Table 2).

In the studies included in this review, changes in circulating FST levels following exercise have been reported under different intensity conditions. Direct within-study comparisons from acute exercise studies suggest that exercise intensity may influence FST responses under some conditions; however, the observed response patterns differ across studies. Some studies reported larger increases under higher-intensity conditions, whereas one study observed greater responses under lower-load resistance exercise conditions. In contrast, chronic exercise evidence remains limited and is primarily derived from single-intensity interventions. Given the limited number of studies, the available evidence should be interpreted with caution.

**Table 2:** Effects of exercise intensity on circulating levels of hepatokines.

Exerkine	Publication Year	Study Population	Exercise Type	Exercise Intensity	Frequency and Duration	Time of Blood Sample	Changes in Concentration	Reference
**Acute Exercise**								
FGF21	2024	9 males; Mean age: 24.0 ± 0.4 years; Baseline fitness levels: untrained	HIIT; VICE; MICE	MICE: (55% HRR); VICE: (85% HRR); HIIT: (all out)	MICE, VICE: Single session, 30 min; HIIT: four bouts of 30 s exercise with 4 min rest intervals	Baseline and immediately after the exercise session as well as 30, 90 min post-exercise.	HIIT: ~75% increase (at 30 min post-exercise); VICE: ~40% increase (at 30 min post-exercise); MICE: no significant change	[54]
	2018	10 males; Mean age: 24 ± 1 years; Baseline fitness levels: trained	HICE; RT	HICE: (70% VO_2_peak); RT: High (90–95% 10RM)	Single session; HICE: 60 min; RT: 58–59 min	Baseline and after the exercise at 15, 30, 60, 90, 120 and 180 min.	HICE: ~261% increase (at 60 min post-exercise); RT: No significant change	[73]
	2019	10 males; Mean age: 26 ± 2 years; Baseline fitness levels: untrained	HICE; MICE	HICE: (75% VO_2_peak); MICE: (55% VO_2_peak)	Single session; HICE: 42 ± 6 min; MICE: 42 ± 6 min	Baseline and at 0, 1, 2, 4, and 7 h post-exercise.	HICE: ~225% increase (at 1 h post-exercise); MICE: ~13% increase (at 1 h post-exercise);	[72]
	2019	14 males; Mean age: 23 ± 1 years; Baseline fitness levels: untrained	HICE; MICE	HICE: (85 ± 8% VO_2_max); MICE: (52 ± 14% VO_2_max)	Single session; HICE: 45 min; MICE: 45 min	Baseline, immediately after the training, and 1, 3, 24, 48, and 72 h after each session.	HICE: ~194% increase (at 1 h post-exercise); MICE: ~55% increase (at 24 h post-exercise)	[71]
	2018	17 males; Mean age: 23 ± 3 years; Baseline fitness levels: untrained	RT	Moderate: (70–75% 1RM)	Single session; 50 min	Baseline and 0, 1, 3, 24, 48, and 72 h post-exercise.	~120% increase (at 24 h post-exercise)	[74]
FST	2019	14 males; Mean age: 23 ± 1 years; Baseline fitness levels: untrained	HICE; MICE	HICE: (85 ± 8% VO_2_max); MICE: (52 ± 14% VO_2_max)	Single session; HICE: 45 min; HICE: 45 min	Baseline, immediately after the training, and 1, 3, 24, 48, and 72 h after each session.	HICE: ~259% increase (at 3 h post-exercise); MICE: ~141% increase (at 3 h post-exercise)	[71]
	2019	10 males; Mean age: 26 ± 2 years; Baseline fitness levels: untrained	HICE; MICE	HICE: (75% VO_2_peak); MICE: (55% VO_2_peak)	Single session; HICE: 42 ± 6 min; MICE: 42 ± 6 min	Baseline and at 0, 1, 2, 4, and 7 h post-exercise.	HICE: ~43% increase (at 4 h post-exercise); MICE: ~30% increase (at 2 h post-exercise)	[72]
	2018	17 males; Mean age: 23 ± 3 years; Baseline fitness levels: untrained	RT	Moderate: (70–75% 1RM)	Single session; 50 min	Baseline and 0, 1, 3, 24, 48, and 72 h post-exercise.	~90% increase (at 24 h post-exercise)	[74]
	2022	10 males; Mean age: 23.2 ± 4.68 years; Baseline fitness levels: trained	RT	High: (80% 1RM); Low: (50% 1RM)	Single session; To volitional failure	Baseline and at 3 and 24 h post-exercise.	Low: ~34.8% increase (at 24 h post-exercise); High: ~22.8% increase (at 24 h post-exercise)	[61]
**Chronic exercise**								
FGF21	2019	12 females; Mean age: 58 ± 5 years; Baseline fitness levels: untrained	RT	Low: (55% 1RM)	Three times per week; 8 weeks	Baseline and post-training.	~10.8% increase	[75]
FST	2020	12 females; Mean age: 23.58 ± 3.84 years; Baseline fitness levels: untrained	RT	Moderate: (65–80% 1RM)	Three times per week; 8 weeks	48 h before training and 48 h after the last training.	~49.15% increase	[65]

Abbreviations: HICE: High-Intensity Continuous Exercise; HIIT: High-Intensity Interval Training; HRR: Heart Rate Reserve; MICE: Moderate-Intensity Continuous Exercise; RT: Resistance Training; RM: Repetition Maximum; VICE: Vigorous-Intensity Continuous Exercise; VO_2_max: Maximal Oxygen Uptake; VO_2_peak: Peak Oxygen Uptake; “~” indicates approximate percentage changes reported in the original studies.

### 4.4. How Exercise Intensity Modulates Adipokine Responses

Adipokines are derived from white adipose tissue and include bioactive peptides, proteins, and immune molecules. Different intensities of exercise can regulate the levels of adipokines such as leptin, adiponectin, and apelin, thus influencing various physiological functions.

#### 4.4.1. Leptin

Leptin is primarily secreted by adipose tissue and released into the bloodstream, where it acts on the arcuate nucleus of the hypothalamus to regulate appetite and satiety, thereby influencing feeding behavior and energy balance [80,81]. In addition, leptin is involved in insulin signaling, promotes lipolysis, reduces striglyceride levels, and improves insulin resistance [82]. Leptin is also closely related to exercise performance by enhancing exercise capacity under conditions of adequate energy availability through modulation of muscle energy metabolism and facilitation of glucose uptake [83,84].

Evidence regarding acute exercise and circulating leptin responses is limited in the studies included in this review. Lakhdar et al. reported that exhaustive exercise induced an acute decrease in circulating leptin levels (~21.4%) immediately after exercise [85] (Table 3). Because only a single exercise intensity was examined in this study, no conclusions regarding the influence of exercise intensity on acute leptin responses can be drawn.

Chronic exercise interventions included in this review report reductions in circulating leptin levels following training, although the magnitude of these reductions varies across studies. Some studies examined only a single exercise intensity. A study by Zhang et al. reported a reduction (~27.6%) following 8 weeks of HICT [86]. In the training component of the study by Lakhdar et al., longer-term high-intensity cycling training was associated with a more pronounced reduction in leptin levels (~44.9%) [85]. In addition, low-intensity resistance training was also associated with a decrease in leptin levels (~7.8%) [75]. In contrast, one study reported no significant change following moderate- to low-intensity resistance training [87]. Middelbeek et al. directly compared different exercise intensities within the same intervention and demonstrated that sprint interval training reduced leptin levels to a greater extent (~23.7%) than moderate-intensity exercise (~11.1%) within the same intervention [88] (Table 3).

In summary, based on the studies included in this review, leptin levels tend to decrease following exercise, particularly in chronic exercise interventions, but this response is not observed across all protocols. Direct within-study comparisons from chronic exercise interventions suggest that exercise intensity may influence leptin responses under some conditions, with high-intensity training producing larger reductions than moderate-intensity exercise within the same intervention. However, acute exercise evidence remains limited to a single-intensity study, and no conclusions regarding intensity-related acute leptin responses can currently be drawn. No single study included in this review directly compared three or more exercise intensity levels for leptin. Given the limited acute evidence, variability across exercise protocols, and the presence of studies reporting no significant change, the available findings should be interpreted with caution.

#### 4.4.2. Adiponectin

Adiponectin is a multifunctional protein hormone secreted by adipose tissue that plays a key role in energy metabolism and cardiovascular health [89,90]. It enhances insulin sensitivity, promotes glucose uptake and utilization in skeletal muscle and liver, and inhibits hepatic glucose production, thereby maintaining glucose homeostasis [91]. In addition, adiponectin stimulates fatty acid oxidation, reduces lipid accumulation in the liver and skeletal muscle, and contributes to protection against obesity and type 2 diabetes [92]. It also exerts anti-inflammatory and anti-atherosclerotic effects by improving vascular function and reducing endothelial inflammation [93]. Circulating adiponectin levels are typically inversely associated with obesity and insulin resistance, making it an important biomarker of metabolic health.

Circulating adiponectin levels may change following acute exercise; however, findings vary across studies and exercise intensities. The available acute exercise evidence consists primarily of single-intensity studies rather than direct within-study intensity comparisons. For example, Kraemer et al. reported an immediate increase (~10%) following running at high intensity (79% VO_2_max) [94], while Mallardo et al. observed peak concentrations (~18.4% increase) at 24 h after high-intensity exercise [95]. In contrast, one study reported no significant change in adiponectin levels following moderate-intensity endurance exercise (50% VO_2_peak) [96] (Table 3).

Chronic exercise interventions included in this review show variable findings across different intensity conditions. Similar to the acute exercise studies, the available chronic evidence is derived from single-intensity interventions rather than direct within-study intensity comparisons. In one study, a four-week HIIT program was associated with an increase in adiponectin levels (~10.7%) [97]. In contrast, a long-term moderate-intensity intervention reported no significant change [98], and low-intensity resistance training similarly did not significantly alter adiponectin levels over an extended period [99] (Table 3).

Based on the studies included in this review, changes in circulating adiponectin levels have been reported following both acute and chronic exercise. These findings vary across studies and exercise intensity conditions. In some studies, increases in adiponectin have been observed following high-intensity exercise, whereas moderate- and low-intensity interventions have shown no changes. Based on observations across the included studies, exercise intensity may influence adiponectin responses, although findings differ across intensity conditions and study designs. However, these observations are derived from indirect comparisons across separate studies rather than direct within-study intensity comparisons. Because these findings were obtained using different exercise modalities, participant characteristics, intervention durations, and sampling protocols, caution is warranted when interpreting potential intensity-related differences across studies.

#### 4.4.3. Apelin

Apelin is an adipokine originally identified in adipose tissue and is also expressed in other tissues, including skeletal muscle and the cardiovascular system [100,101]. It is an endogenous ligand of the APJ receptor and plays a multifaceted role in metabolic and cardiovascular regulation [102,103]. Apelin contributes to glucose and lipid metabolism by improving insulin sensitivity, promoting glucose uptake, and regulating energy homeostasis [104]. In the cardiovascular system, it participates in vascular relaxation, angiogenesis, cardiac contractility, and blood pressure regulation [101,103]. In addition, apelin has been implicated in skeletal muscle adaptation, mitochondrial metabolism, exercise capacity, and adipose tissue remodeling [105,106,107]. Apelin may contribute to inter-organ communication during exercise via endocrine and paracrine actions and has been proposed as a potential exercise-responsive exerkine [14,28].

Acute exercise studies included in this review suggest that circulating apelin levels may change following exercise; however, findings vary across studies. Kon et al. reported that sprint interval exercise induced a significant increase in apelin levels (~31%) immediately after exercise [53]. Similarly, Ligetvári et al. observed increases in apelin isoforms following maximal cardiopulmonary exercise testing, with apelin-13 increasing by approximately 16% and apelin-36 by approximately 149% immediately post-exercise [106]. In contrast, Son et al. reported no significant change in circulating apelin levels following a single bout of exhaustive exercise [108]. Likewise, Waller et al. observed no significant changes in apelin levels following exercise performed at high intensity [109]. Notably, all acute exercise studies included in this review examined only a single exercise intensity, and no direct within-study intensity comparisons were identified.

Chronic exercise interventions included in this review suggest that apelin levels may increase following training. Fujie et al. reported that an 8-week high-intensity endurance exercise program increased circulating apelin levels by approximately 127% [110]. In a similar study, Fujie et al. also observed an increase in apelin levels (~116%) following an 8-week high-intensity aerobic training intervention [111]. The chronic exercise studies included in this review were also limited to single-intensity interventions, and no direct comparisons across different exercise intensities were available.

Based on the studies included in this review, apelin responses to exercise appear to differ between acute and chronic conditions. Acute exercise studies suggest variable findings, with some reporting increases in apelin levels and others observing no significant changes. The limited number of chronic exercise studies included in this review report increases in circulating apelin levels following training. Notably, the studies included in this review were conducted under high-intensity exercise conditions. The absence of comparisons across different intensity levels makes it difficult to determine whether apelin responses are associated with exercise intensity.

The concentration of studies included in this review under high-intensity exercise conditions is also related to the inclusion criteria of the present review. This review focused exclusively on healthy populations and did not include studies involving individuals with chronic metabolic diseases. Existing studies investigating the effects of moderate- and low-intensity exercise on apelin responses have been conducted primarily in populations with obesity, diabetes, and other metabolic disorders and therefore did not meet the inclusion criteria of the present review. As a result, the studies included in this review were largely limited to high-intensity exercise conditions, which restricted both direct and indirect comparisons across exercise intensities and made it difficult to determine whether apelin responses are associated with exercise intensity.

**Table 3:** Effects of exercise intensity on circulating levels of adipokines.

Exerkine	Publication Year	Study Population	Exercise Type	Exercise Intensity	Frequency and Duration	Time of Blood Sample	Changes in Concentration	Reference
**Acute exercise**								
Leptin	2013	8 males; Mean age: 20.7 ± 4.8 years; Baseline fitness levels: trained	HICE	High: (-)	Maximal Exercise, Single session;	Baseline, at the end and after 30, 60 min of recovery.	~21.4% increase (immediately after exercise)	[85]
Adiponectin	2024	15 males; Mean age: 25.3 ± 4.1 years; Baseline fitness levels: trained	HICE	High: (exhaustive exercise)	Single session; To exhaustive	Baseline, at 15 min and 24 h post-exercise.	~18.4% increase (at 24 h post-exercise)	[95]
	2003	6 males; Mean age: 23 ± 1.34 years; Baseline fitness levels: untrained	HICE	High: (79% VO_2_max)	Single session; 30 min	Baseline and immediately after the exercise session as well as 30 min post-exercise.	~10% increase (immediately post-exercise)	[94]
	2008	8 males; Mean age: 24.9 ± 1.8 years; Baseline fitness levels: untrained	MICE	Moderate: (50% VO_2_peak)	Single session; 60 min	Baseline, 20, 40, 60 min after the start of exercise, and 30 min post-exercise.	No significant change	[96]
Apelin	2021	8 males; Mean age: 20.3 ± 0.6 years; Baseline fitness levels: untrained	SIT	High: (maximal efforts)	Single session; Four bouts of 30-s all-out sprints	Baseline and immediately after the exercise session as well as 15, 30 and 120 min post-exercise.	~31% increase (immediately post-exercise)	[53]
	2019	8 males; Mean age: 37.38 ± 9.75 years; Baseline fitness levels: untrained	A single bout of exhaustive exercise.	High: (exhaustive exercise)	Single session; To exhaustive	Baseline and immediately after the exercise session as well as 15, 30 min post-exercise.	No significant change	[108]
	2019	7 males and 5 females; Mean age: 22.8 ± 2.9 years; Baseline fitness levels: untrained	HICE	High: (maximal VO_2_max); (70–75% VO_2_max)	Single session; maximal VO_2_max: 7.21 ± 0.3 min; 70–75% VO_2_max: 30 min	Baseline and immediately after the exercise session as well as 1, 24 h post-exercise.	No significant change	[109]
	2023	58 males; Mean age: 22.9 ± 4.7 years; Baseline fitness levels: trained	HICE	High (maximal cardiorespiratory exercise)	Single session; 11.2 ± 1.5 min	Baseline, immediately after the exercise, and 30 min post-exercise.	Apelin-13: ~16% increase (immediately post-exercise); Apelin-36: ~149% increase (immediately post-exercise)	[106]
**Chronic exercise**								
Leptin	2021	22 males; Mean age: 48 ± 5 years; Baseline fitness levels: untrained	SIT; MICE	SIT: High: (all out); MICE: (60% VO_2_peak)	SIT: six times per two weeks; MICE: six times per two weeks	Baseline and 48 h after the last exercise session.	SIT: ~23.7% decrease; MICE: ~11.1% decrease	[88]
	2013	8 males; Mean age: 20.7 ± 4.8 years; Baseline fitness levels: trained	HICE	High: (-)	Intense cycling training, six months	Baseline, at the end and after 30, 60 min of recovery.	~44.9% decrease	[85]
	2024	14 females; Mean age: 22.1 ± 1.6 years; Baseline fitness levels: untrained	HICT	High: (80–82% HRmax)	Three times per week; 8 weeks	Baseline and post-training.	~27.6% decrease	[86]
	2021	20 males; aged 35 to 60 years; Baseline fitness levels: untrained	RT	Moderate to low: (50–70% 1RM)	Three times per week; 12 weeks	Baseline, the sixth week and the twelfth week.	No significant change	[87]
	2019	12 females; Mean age: 58 ± 5 years; Baseline fitness levels: untrained	RT	Low: (55% 1RM)	Three times per week; 8 weeks	Baseline and post-training.	~7.8% decrease	[75]
Adiponectin	2013	5 males and 2 females; Mean age: 19 ± 1.2 years; Baseline fitness levels: trained	HIIT	High: (70% HRmax)	Twice per week; 4 weeks	Baseline and post-training.	~10.7% increase	[97]
	2023	57 females; Mean age: 58.7 ± 4.9 years; Baseline fitness levels: untrained	MICE	Moderate: (3 MET-hours)	Up to 1 h per day; 24 months	Baseline and post-exercise.	No significant change	[98]
	2007	12 males; Mean age: 20.8 ± 3.0 years; Baseline fitness levels: trained	RT	Low: (50% 1RM)	24 weeks	Every four weeks.	No significant change	[99]
Apelin	2022	7 males and 10 females; Mean age: 65.5 ± 2.0 years; Baseline fitness levels: untrained	HICE	High: (60–70% VO_2_peak)	Three times per week; 8 weeks	Every two weeks.	~127% increase	[110]
	2014	7 males and 11 females; Mean age: 66.4 ± 2.1 years; Baseline fitness levels: untrained	HICE	High: (60–70% VO_2_peak)	Three times per week; 8 weeks	Baseline and 48 h after the last exercise session.	~116% increase	[111]

Abbreviations: HICE: High-Intensity Continuous Exercise; HICT: High-Intensity Circuit Training; HIIT: High-Intensity Interval Training; MICE: Moderate-Intensity Continuous Exercise; RM: Repetition Maximum; RT: Resistance Training; SIT: Sprint Interval Training. VO_2_max: Maximal Oxygen Uptake; VO_2_peak: Peak Oxygen Uptake; “(-)” indicates that the specific numerical threshold for exercise intensity was not reported in the original study; “~” indicates approximate percentage changes reported in the original studies.

### 4.5. How Exercise Intensity Modulates Brain-Derived Neurotrophic Factor (BDNF) Responses

Neurotrophic factors are a family of molecules that regulate the development, survival, and function of neurons in both the central and peripheral nervous systems. This family consists of various factors, with a primary focus on BDNF in this article.

BDNF is a member of the neurotrophin family that is widely distributed in both the central and peripheral nervous systems [112]. It promotes neuronal survival, differentiation, and the growth of axons and dendrites and plays a central role in synaptic plasticity, learning, and memory formation [113]. BDNF exerts its biological effects primarily through binding to its high-affinity receptor, tropomyosin receptor kinase B (TrkB), activating signaling pathways involved in neuronal metabolism and anti-apoptotic processes [114]. In peripheral tissues, BDNF also influences skeletal muscle metabolism, cardiovascular function, and immune regulation [115,116], making it an important link between exercise and systemic physiological adaptation.

Acute exercise studies suggest that circulating BDNF levels may increase following exercise; however, findings vary across studies and exercise intensity conditions. Several studies included direct within-study comparisons of different exercise intensities. For example, Reycraft et al. demonstrated progressively greater increases in BDNF with increasing exercise intensity, with sprint interval exercise producing the highest concentrations immediately after exercise [117]. Similarly, Ji et al. reported that HIIT increased BDNF by approximately 55% immediately post-exercise, whereas moderate-intensity exercise did not elicit significant changes [54]. One study further reported that high-intensity exercise induced a marked increase in BDNF (~275%), with peak concentrations observed approximately 60 min post-exercise, whereas moderate-intensity exercise did not result in measurable changes [118]. These direct within-study comparisons suggest that exercise intensity may influence acute BDNF responses under some conditions. In addition to the direct comparison studies, single-intensity studies have also reported exercise-induced increases in BDNF levels. For example, high-intensity interval protocols have been associated with rapid increases in BDNF (~236%) [119]. Furthermore, one study reported that moderate-intensity exercise induced modest increases in BDNF (~13–16%) [120], whereas another study observed a more pronounced increase (~87%), with peak concentrations occurring approximately 60 min post-exercise [121] (Table 4).

Chronic exercise interventions included in this review are limited and show variable findings across intensity conditions. The available chronic evidence consists primarily of single-intensity interventions rather than direct within-study intensity comparisons. One study reported no significant changes in BDNF circulating levels following a moderate-intensity endurance training program [122]. In contrast, low-intensity resistance training was associated with an increase in BDNF levels (~22.9%) after 12 weeks of intervention [123] (Table 4).

Based on the studies included in this review, increases in circulating BDNF levels have been frequently observed following acute exercise, although findings vary across studies and exercise intensity conditions. Direct within-study comparisons from acute exercise studies suggest that different exercise intensities may be associated with different BDNF response patterns under some conditions, with some studies reporting larger increases under higher-intensity exercise conditions. In contrast, chronic exercise evidence remains limited and is currently derived from single-intensity interventions. Therefore, the available evidence suggests that exercise intensity may influence BDNF responses under some conditions, although the findings should be interpreted cautiously.

**Table 4:** Effects of exercise intensity on circulating levels of brain-derived neurotrophic factor (BDNF).

Publication Year	Study Population	Exercise Type	Exercise Intensity	Frequency and Duration	Time of Blood Sample	Changes in Concentration	Reference
**Acute exercise**							
2019	8 males; Mean age: 23.1 ± 3.0 years; Baseline fitness levels: untrained	MICE; VICE; SIT	MICE: (65% VO_2_max); VICE: (85% VO_2_max); SIT, high: (maximal effort)	Single session; 30 min	Baseline and immediately after the exercise session as well as 30 min and 90 min post-exercise.	Increased in an intensity-dependent manner, SIT induced the highest BDNF concentration, ~190% increase (immediately post-exercise)	[117]
2024	9 males; Mean age: 24.0 ± 0.4 years; Baseline fitness levels: untrained	HIIT; VICE; MICE	MICE (55% HRR); VICE (85% HRR); HIIT (all out)	MICE, VICE: Single session; 30 min; HIIT: four bouts of 30 s exercise with 4 min rest intervals	Baseline and immediately after the exercise session as well as 30 min and 90 min post-exercise.	HIIT: ~55% increase (immediately post-exercise); VICE: ~25% increase (immediately post-exercise); MICE: no significant change	[54]
2022	20 males; Mean age: 29.1 ± 5.8 years; Baseline fitness levels: trained	HIIT	High: (100% VO_2_peak)	Single session; 10 × 1 min	Baseline and post-exercise.	~236% increase (post-exercise)	[119]
2021	20 males; Mean age: 60.8 ± 1.7 years; Baseline fitness levels: trained	RT; MICE	RT, moderate: (65–70% 1RM); MICE: (65–70% HRmax)	Single session; 45 min	Baseline and post exercise at 10 min.	RT: ~16% increase (post-exercise at 10 min); MICE: ~13% increase (post-exercise at 10 min)	[120]
2024	14 males; Mean age: 41.0 ± 5.8 years; Baseline fitness levels: trained	RT	High: (80% 1RM); Moderate: (60% 1RM)	Single session; four groups	Baseline and immediately after the training session as well as 60 min post-exercise.	High: ~275% increase (post-exercise); Moderate: no significant change	[118]
2020	12 young men; Mean age: 25.3 ± 5.9 years; Baseline fitness levels: trained	RT	Moderate: (65% 1RM)	Single session; five groups, ten repetitions per group	Baseline, post-exercise immediately, 1 h post-exercise, and 2 h post-exercise.	~87% increase (immediately post-exercise)	[121]
**Chronic exercise**							
2023	7 males and 13 females; Mean age: 22.26 ± 1.68 years; Baseline fitness levels: untrained	MICE	Moderate: (55–70% HRmax)	Five times per week; 4 weeks	One day before and after the exercise.	No significant change	[122]
2015	9 males; Mean age: 68.05 ± 6.4 years; Baseline fitness levels: untrained	RT	Low: (20–40% 1RM)	Three times per week; 12 weeks	Baseline and after 12 weeks (24 h–48 h after the last training).	~22.9% increase	[123]

Abbreviations: HIIT: High-Intensity Interval Training; HRR: Heart Rate Reserve; MICE: Moderate-Intensity Continuous Exercise; RM: Repetition Maximum; RT: Resistance Training; SIT: Sprint Interval Training; VICE: Vigorous-Intensity Continuous Exercise; VO_2_max: Maximal Oxygen Uptake; VO_2_peak: Peak Oxygen Uptake; “~” indicates approximate percentage changes reported in the original studies.

## 5. Integrated Interpretation of Exerkine Responses to Exercise Intensity

**Figure 2:**
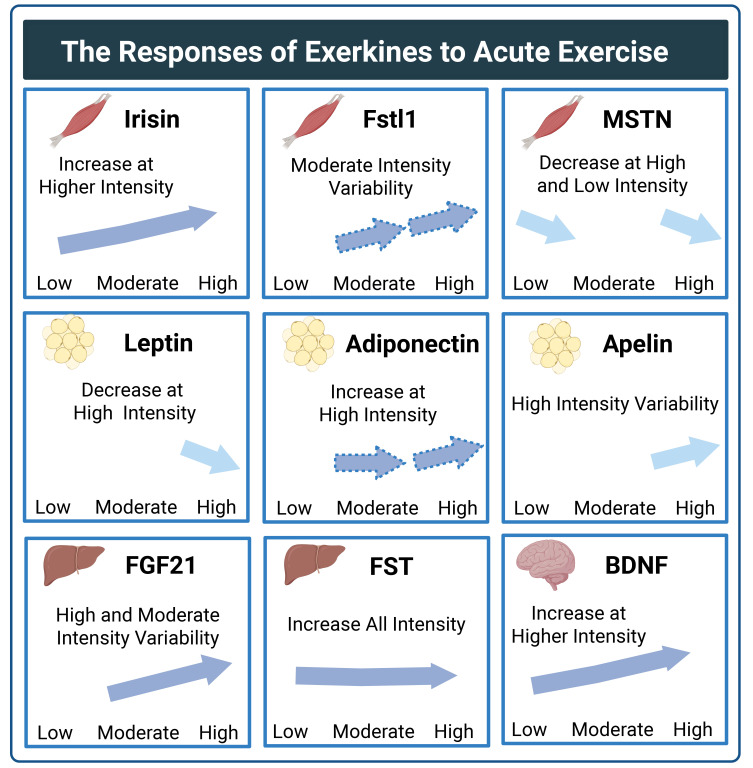
Responses of circulating exerkines to acute exercise across different exercise intensities. Arrow direction indicates the overall trend in circulating exerkine levels (upward for increase and downward for decrease). Continuous arrows spanning multiple exercise intensities indicate that changes in exerkine levels were observed across the indicated intensity range. Upward-sloping continuous arrows indicate greater responses with increasing exercise intensity, whereas horizontal arrows spanning all intensities indicate changes observed across low-, moderate-, and high-intensity exercise conditions. Horizontal arrows at a single intensity indicate no measurable changes under that specific intensity condition. Solid line arrows represent findings supported by ≥2 direct within-study intensity comparisons. Dashed arrows represent observations derived primarily from indirect comparisons across separate studies. Lighter-colored single arrows indicate findings based only on single-intensity studies.

Responses the nine exerkines discussed in this narrative review differ between acute and chronic exercise conditions, and also vary across individual studies. Based on the acute exercise studies included in this review, changes in circulating exerkines have been reported for several molecules. The number of studies and the range of exercise intensities examined differ substantially across exerkines. For example, for BDNF and irisin, some studies included direct within-study comparisons across different intensities and reported larger increases under higher-intensity conditions, although such findings are not uniform across studies and exercise conditions. For FGF21, FST, and Fstl1, a limited number of direct within-study comparisons suggest that exercise intensity may be associated with differences in the magnitude of responses under some conditions; however, these observations are based on a small number of studies and are not uniform across studies and exercise conditions. For MSTN, the acute studies included in this review provide limited comparisons across different intensity levels, with available evidence primarily directly comparing high- and low-intensity conditions rather than a full range of intensities. For leptin, acute exercise included in this review is limited to a single study, which reported a decrease following high-intensity exercise. For adiponectin, findings on the effects of acute exercise are based on a small number of single-intensity studies, including increases reported under high-intensity conditions and no changes under moderate intensity. Studies on the effects of acute exercise on apelin levels included in this review were primarily conducted under high-intensity conditions, with some reporting increases and others observing no significant change (Figure 2). Notably, only a small number of studies included direct within-study comparisons across three or more exercise intensity levels within the same experimental design (Table 5), highlighting the limited availability of robust dose–response evidence in the current literature. In addition, all studies that included direct within-study intensity comparisons in this review have been summarized in Supplementary Tables S3 and S4.

Chronic exercise studies included in this review are limited in number and show heterogeneous findings across exerkines. For several molecules, including irisin, FGF21, FST, apelin, BDNF, and adiponectin, increases in circulating levels have been reported following training in some studies, whereas other studies have reported no measurable changes. For MSTN and leptin, reductions in circulating levels have been observed in some chronic interventions, while no significant changes have also been reported in other studies. Based on the studies included in this review, the number of chronic exercise studies remains limited, and direct within-study comparisons across different exercise intensity levels are scarce, making it difficult to determine the role of exercise intensity in long-term adaptations.

Taken together, based on the studies included in this review, circulating exerkine levels respond to exercise under acute and chronic conditions. In acute exercise, a limited number of studies suggest that exercise intensity may influence the magnitude of responses for some exerkines; however, these findings are not uniform across studies and are not available for all molecules. In chronic exercise, the role of exercise intensity remains unclear due to the small number of studies and the lack of multi-intensity comparisons. Therefore, given the limited number of studies, variability in study designs, and differences in exercise protocols, these findings should be interpreted with caution.

## 6. Limitations

Several limitations should be acknowledged when interpreting the findings discussed in this review. This article was not designed as a systematic review, and the literature search and study selection were not conducted according to PRISMA guidelines; accordingly, no formal risk of bias assessment or quantitative evaluation of study quality was performed. A major constraint is the lack of direct comparisons across multiple exercise intensities within the same experimental framework, as most available studies employ single-intensity protocols. This limits the ability to establish a clear and comparable intensity–response relationship across different exerkines and means that conclusions regarding the role of exercise intensity are largely based on indirect comparisons between studies. In addition, substantial heterogeneity exists in study designs, including differences in exercise modalities such as endurance and resistance exercise, and differences in participant characteristics, including age, training status, metabolic condition, and nutritional status (e.g., fasted vs. fed state). These factors may independently influence exerkine responses and complicate the interpretation of intensity-specific effects. Furthermore, the definition and quantification of exercise intensity are not uniform across studies, with indicators such as percentage of maximal oxygen uptake, percentage of maximal heart rate, and percentage of one-repetition maximum used interchangeably, and with thresholds for “high intensity” varying across studies (e.g., 70% vs. 85% HRmax), thereby limiting comparability and potentially obscuring true intensity-related patterns. Differences in the timing of blood sampling following exercise also represent an additional source of variability, as peak exerkine responses may occur at different post-exercise time points. Moreover, many of the studies included in this review have relatively small sample sizes, and the number of available studies for each individual exerkine remains limited. Finally, potential publication bias may exist because this review did not employ a systematic search strategy; studies reporting no intensity-related differences or null results were likely under-sampled relative to studies reporting positive findings. These limitations may partly explain the variability observed in intensity-related responses across different exerkines and should be considered when interpreting the role of exercise intensity in their regulation.

## 7. Conclusions

Based on the studies included in this review, the role of exercise intensity in exerkine responses appears to be complex and context-dependent. In acute exercise studies, some direct within-study comparisons suggest that higher exercise intensities may be associated with greater changes in circulating exerkine levels under some conditions. For example, studies on irisin and BDNF reported larger increases under higher-intensity exercise conditions within the same experimental design. For FGF21, FST, Fstl1, MSTN, leptin, and adiponectin, some studies also reported different response magnitudes across exercise intensity conditions; however, these findings were limited by the small number of direct comparisons and inconsistent results across studies. In contrast, the current evidence for apelin remains insufficient to determine whether exercise intensity exerts a consistent effect. Studies examining chronic exercise included in this review do not provide asufficient basis for evaluating the role of exercise intensity, as comparisons across different intensity levels are limited. Overall, the available evidence suggests that exercise intensity may influence exerkine responses under some conditions, particularly during acute exercise; however, the current evidence remains limited and inconsistent across different exerkines and exercise protocols. It should also be noted that these conclusions are based on a subset of exerkines included in this review and may not be generalizable to all exercise-responsive molecules. Furthermore, because this review did not employ a systematic search strategy, the findings should be interpreted cautiously, and the absence of a systematic approach should be considered an important limitation of the present review.

## Figures and Tables

**Table 5:** Studies including direct within-study comparisons across three or more exercise intensities.

Exercise Intensity	Study Population	Frequency and Duration	Effects on Circulating Exerkine Levels	Reference
MICE: (65% VO_2_max); VICE: (85% VO_2_max); SIT, high: (maximal effort)	8 males; Mean age: 23.1 ± 3.0 years; Baseline fitness levels: untrained	Single session; 30 min	For BDNF: SIT > VICE > MICE	[117]
MICE (55% HRR); VICE (85% HRR); HIIT (all out)	9 males; Mean age: 24.0 ± 0.4 years; Baseline fitness levels: untrained	MICE, VICE: Single session; 30 min; HIIT: four bouts of 30-s exercise with 4 min rest intervals	For BDNF: HIIT > VICE >MICE; For FGF21: HIIT > VICE > MICE; For Fstl1: HIIT > VICE > MICE	[54]

Abbreviations: HIIT: High-Intensity Interval Training; MICE: Moderate-Intensity Continuous Exercise; SIT: Sprint Interval Training; VICE: Vigorous-Intensity Continuous Exercise.

## Data Availability

No new data were created or analyzed in this study.

## Supplementary Materials

The following supporting information can be downloaded at https://www.mdpi.com/article/10.3390/biom16060852/s1, Figure S1: Flow diagram of literature identification and study selection for the present narrative review; Table S1: Representative examples of exercise-responsive molecules not included in the present narrative review and reasons for non-inclusion; Table S2: Literature Search Strategy and Eligibility Criteria; Table S3: Acute exercise studies including direct within-study comparisons; Table S4: Chronic exercise studies including direct within-study comparisons; Table S5: Included studies investigating the effects of exercise intensity on circulating Irisin levels; Table S6: Included studies investigating the effects of exercise intensity on circulating Fstl1 levels; Table S7: Included studies investigating the effects of exercise intensity on circulating MSTN levels; Table S8: Included studies investigating the effects of exercise intensity on circulating FGF21 levels; Table S9: Included studies investigating the effects of exercise intensity on circulating FST levels; Table S10: Included studies investigating the effects of exercise intensity on circulating Leptin levels; Table S11: Included studies investigating the effects of exercise intensity on circulating Adiponectin levels; Table S12: Included studies investigating the effects of exercise intensity on circulating Apelin levels; Table S13: Included studies investigating the effects of exercise intensity on circulating BDNF levels.

## Abbreviations

The following abbreviations are used in this manuscript:

AT	Anaerobic Threshold
BDNF	Brain-derived neurotrophic factor
FGF21	Fibroblast growth factor 21
FNDC5	Fibronectin type III domain-containing protein 5
FST	Follistatin
Fstl1	Follistatin-like protein 1
HICE	High-Intensity Continuous Exercise
HICT	High-Intensity Circuit Training
HIIT	High-Intensity Interval Training
HRmax	Maximum heart rate
HRR	Heart rate reserve
IL-6	Interleukin-6
LICE	Low-Intensity Continuous Exercise
METs	Metabolic equivalents
MICE	Moderate-Intensity Continuous Exercise
MSTN	Myostatin
PGC1-1α	Peroxisome proliferator-activated receptor gamma coactivator-1α
RT	Resistance Training
SIT	Sprint Interval Training
TGF-β	Transforming growth factor-β
TrkB	Tropomyosin receptor kinase B
VICE	Vigorous-Intensity Continuous Exercise
VO_2_max	Maximal oxygen uptake
VO_2_peak	Peak oxygen uptake
WHO	World Health Organization
