# Supplementary material for: Exercise Intensity and Circulating Exerkine Responses: A Narrative Review of Selected Molecules
Source: Biomolecules. 2026 Jun 10;16(6):852. doi: 10.3390/biom16060852 (PMC13297134; doi:10.3390/biom16060852)
Supplement: Supplementary file 1 [file biomolecules-16-00852-s001.zip › biomolecules-4289006-supplementary.pdf]

# Supplementary Materials

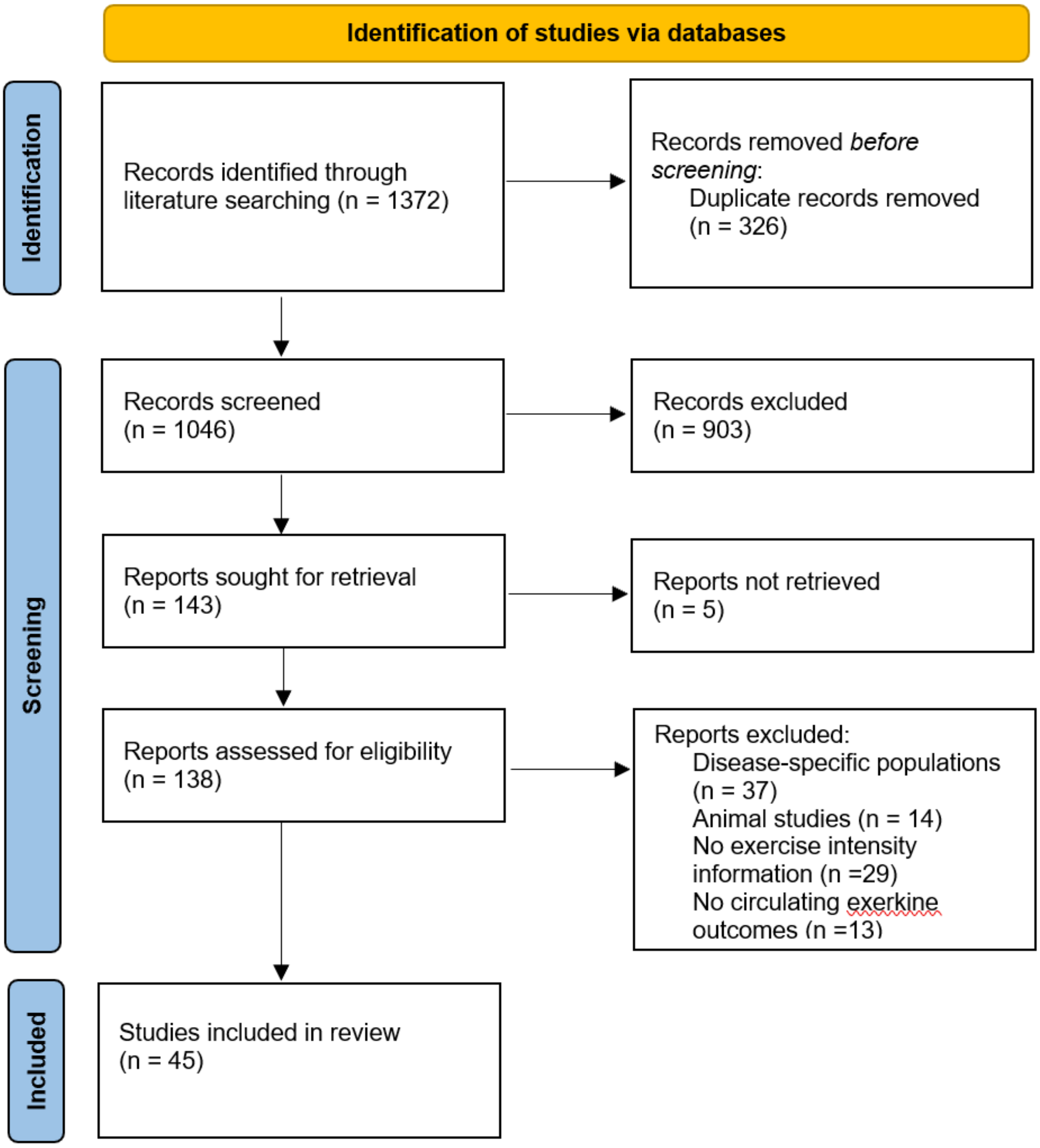


**Supplementary Figure S1.** Flow diagram of literature identification and study selection for the present narrative review.

**Supplementary Table S1.** Representative examples of exercise-responsive molecules not included in the present narrative review and reasons for non-inclusion.

| Exerkine | Reason for non-inclusion |
|---|---|
| Decorin | Limited available exercise studies and heterogeneous exercise protocols. |
| Cathepsin B | |
| Omentin | Existing studies mainly focused on clinical or disease populations, which did not meet the inclusion criteria of the present review. |
| β-minoisobutyric acid | |
| Meteorin-like | Limited available human studies examining exercise intensity-related responses. |
| Growth Differentiation Factor 15 | |

Note: These examples are provided to improve transparency regarding the scope of the present narrative review and should not be interpreted as a comprehensive list of all excluded exercise-responsive molecules. Furthermore, the present review cannot rule out the possibility that other exercise-responsive molecules may exhibit different intensity-response patterns from those discussed in this manuscript.

**Supplementary Table S2.** Literature Search Strategy and Eligibility Criteria.

| **Iteam** | **Description** |
|---|---|
| Review type | Narrative review |
| Databases searched | PubMed and Google Scholar |
| Search period | From database inception to March 2026 |
| Search terms | “exerkines,” “exercise intensity,” “irisin,” “Fstl1,” “follistatin-like protein 1,” “MSTN,” “myostatin,” “FGF21,” “fibroblast growth factor 21,” “FST,” “follistatin,” “leptin,” “adiponectin,” “apelin,” “brain-derived neurotrophic factor,” “BDNF,” “high-intensity exercise,” “moderate-intensity exercise,” “low-intensity exercise,” “resistance training,” and “endurance exercise” |
| Inclusion criteria | Human studies; healthy participants; studies reporting circulating exerkine responses before and after exercise; studies providing information on exercise intensity |
| Exclusion criteria | Animal studies; disease-specific intervention studies; studies without clearly defined exercise intensity; studies without circulating exerkine outcomes |

**Supplementary Table S3.** Acute exercise studies including direct within-study comparisons.

| Exercise Intensity | Study Population | Frequency & Duration | Effects on circulating exerkine levels | Reference |
|---|---|---|---|---|
| MICE:<br>(50% HRR);<br>HIIT:<br>(85%-90% HRR) | 7 males and 7 females;<br>Mean age:<br>17.14 ± 1.66 years;<br>Baseline fitness levels:<br>untrained | MICE:<br>Single session, 35min;<br>HIIT:<br>Single session, 35min;<br>(total session,<br>including rest intervals) | For Irisin: HIIT > MICE | [1] |
| MICE:<br>(50%-55% HRR);<br>HIIT:<br>(70%-75% HRR) | 10 males and 11 females;<br>Mean age:<br>60.62 ± 4.96 years;<br>Baseline fitness levels:<br>untrained | MICE:<br>Single session; 30min<br>HIIT:<br>Single session; 30min<br>(total session,<br>including rest intervals) | For Irisin: HIIT > MICE | [2] |
| HICE:<br>(80% $VO_2max$);<br>LICE:<br>(40% $VO_2max$) | 6 males;<br>Mean age:<br>22.5 ± 1.1 years;<br>Baseline fitness levels:<br>untrained | HICE:<br>Single session, 40min;<br>LICE:<br>Single session, 20min | For Irisin: HICE > LICE | [3] |
| MICE: (-);<br>HIIT: (-) | 15 males;<br>mean age:<br>15.4 ± 0.2 years;<br>and 15 females,<br>mean age:<br>15.4 ± 0.3 years;<br>Baseline fitness levels:<br>trained | MICE:<br>Single session,<br>27 min 37 ± 22 s;<br>HIIT:<br>Single session,<br>32.23 ± 0.47 s | For Irisin: HIIT > MICE | [4] |
| MICE (55% HRR);<br>VICE (85% HRR);<br>HIIT (all out) | 9 males;<br>Mean age:<br>24.0 ± 0.4 years;<br>Baseline fitness levels:<br>untrained | MICE, VICE:<br>Single session; 30min;<br>HIIT: four bouts of<br>30-second exercise<br>with 4-minute<br>rest intervals | For Fstl1: HIIT > VICE >MICE;<br>For FGF21: HIIT > VICE >MICE;<br>For BDNF: HIIT > VICE >MICE | [5] |
| High:<br>(80% 1RM);<br>Low:<br>(50% 1RM) | 10 males;<br>Mean age:<br>23.2 ± 4.68 years;<br>Baseline fitness levels:<br>trained | Single session;<br>To volitional failure | For MSTN: High > Low;<br>For FST: Low > High | [6] |

| | | | | |
|---|---|---|---|---|
| HICE:<br>(70% $VO_2$peak);<br>RT: High<br>(90%-95% 10RM) | 10 males;<br>Mean age:<br>24 ± 1 years;<br>Baseline fitness levels:<br>trained | Single session;<br>HICE:<br>60min;<br>RT:<br>58-59min | For FGF21: HICE > RT | [7] |
| HICE:<br>(75% $VO_2$peak);<br>MICE:<br>(55% $VO_2$peak) | 10 males;<br>Mean age:<br>26 ± 2 years;<br>Baseline fitness levels:<br>untrained | Single session;<br>HICE:<br>42 ± 6min;<br>MICE:<br>42 ± 6min | For FGF21: HICE > MICE;<br>For FST: HICE > MICE | [8] |
| HICE:<br>(85±8% $VO_2$max);<br>MICE:<br>(52±14% $VO_2$max) | 14 males;<br>Mean age:<br>23 ± 1 years;<br>Baseline fitness levels:<br>untrained | Single session;<br>HICE: 45min;<br>MICE: 45min | For FGF21: HICE > MICE;<br>For FST: HICE > MICE | [9] |
| MICE:<br>(65% $VO_2$max);<br>VICE:<br>(85% $VO_2$max);<br>SIT, high:<br>(maximal effort) | 8 males；<br>Mean age:<br>23.1 ± 3.0 years;<br>Baseline fitness levels:<br>untrained | Single session;<br>30min | For BDNF: SIT > VICE > MICE | [10] |
| High:<br>(80% 1RM);<br>Moderate:<br>(60% 1RM) | 14 males;<br>Mean age:<br>41.0 ± 5.8 years;<br>Baseline fitness levels:<br>trained | Single session;<br>four groups | For BDNF: High > Moderate | [11] |

Abbreviations: HICE: High-Intensity Continuous Exercise; HIIT: High-Intensity Interval Training; LICE: Low-Intensity Continuous Exercise; MICE: Moderate-Intensity Continuous Exercise; RT: Resistance training; SIT: Sprint Interval Training; VICE: Vigorous-Intensity Continuous Exercise; "~" indicates approximate percentage changes reported in the original studies.

**Supplementary Table S4.** Chronic exercise studies including direct within-study comparisons.

| **Exercise Intensity** | **Study Population** | **Frequency & Duration** | **Effects on circulating exerkine levels** | **Reference** |
|---|---|---|---|---|
| MICE:<br>(60%-70% HRmax);<br>HIIT:<br>(90% HRmax) | MICE:<br>Three times per week, 12week;<br>HIIT:<br>Three times per week, 2week | MICE:<br>Three times per week, 12week;<br>HIIT:<br>Three times per week, 2week | For MSTN: MICE > HIIT | [12] |
| SIT:<br>High: (all out);<br>MICE:<br>(60% $VO_2$peak) | 22 males;<br>Mean age:<br>48 ± 5 years;<br>Baseline fitness levels: untrained | SIT: six times per two weeks;<br>MICE: six times per two weeks | For Leptin: SIT > MICE | [13] |

Abbreviations: HIIT: High-Intensity Interval Training; MICE: Moderate-Intensity Continuous Exercise; SIT: Sprint Interval Training.

**Supplementary Table S5.** Included studies investigating the effects of exercise intensity on circulating Irisin levels.

| Study ID | Study Population | Exercise Type | Exercise Intensity | Frequency & Duration | Time of Blood Sample | Changes in Concentration (percentage change) | Changes in Concentration (absolute values) | Reference |
|---|---|---|---|---|---|---|---|---|
| **Acute exercise** | | | | | | | | |
| Colpitts 2022 | 7 males and 7 females; Mean age: 17.14 ± 1.66 years; Baseline fitness levels: untrained | HIIT; MICE | MICE: (50% HRR); HIIT: (85%-90% HRR) | MICE: Single session, 35min; HIIT: Single session, 35min; (total session, including rest intervals) | Baseline and during the training at 7, 14, 21, 28 min and immediately post-exercise. | MICE: no significant change; HIIT: ~81.0% increase (during the training) | MICE: baseline: 20.65 ± 8.05 ng/mL, post-exercise: not available; HIIT: baseline: 19.32 ± 13.16 ng/mL, post-exercise: not available | [1] |
| Tsai 2021 | 10 males and 11 females; Mean age: 60.62 ± 4.96 years; Baseline fitness levels: untrained | HIIT; MICE | MICE: (50%-55% HRR); HIIT: (70%-75% HRR) | MICE: Single session; 30min HIIT: Single session; 30min (total session, including rest intervals) | Baseline and post-exercise. | MICE: no significant increase; HIIT: ~8.4% increase (post-exercise) | MICE: baseline: 629.77.19 ± 111.76 ng/mL, post-exercise: 657.81 ± 113.25 ng/ml; HIIT: baseline: 622.23 ± 151.81 ng/mL, post-exercise: 674.32 ± 150.08 ng/ml | [2] |
| Tsuchiya 2014 | 6 males; Mean age: 22.5 ± 1.1 years; Baseline fitness levels: untrained | HICE; LICE | HICE: (80% $VO_2max$); LICE: (40% $VO_2max$) | HICE: Single session, 40min; LICE: Single session, 20min | Baseline and immediately after exercise, at 3, 6, and 19 hours post-exercise. | HICE: ~18% increase 6 hours and ~23% increase 19 hours post-exercise; LICE: ~38% decrease (immediately post-exercise) | HICE: baseline: 122.4 ± 42.6 ng/mL, post-exercise (6 h): 136.2 ± 28.4 ng/ml, post-exercise (19 h): 138.6 ± 25.5 ng/ml; LICE: baseline: 175.0 ± 25.2 ng/mL, post-exercise (0 h): 107.0 ± 27.2 ng/ml | [3] |
| Huh 2014 | 15 males; mean age: 15.4 ± 0.2 years; and 15 females, mean age: 15.4 ± 0.3 years; Baseline fitness levels: trained | HIIT; MICE | MICE: (-); HIIT: (-) | MICE: 27 min 37 ± 22 s; HIIT: 32.23 ± 0.47 s | Baseline, immediately (in less than 5 min), 1 hour, and 24 hours post-exercise. | MICE: no significant change; HIIT: ~30% increase (immediately post-exercise) | MICE: baseline: 91.4 ± 5.0 ng/mL, post-exercise: not available; HIIT: baseline: 94.2 ± 8.0 ng/mL, post-exercise: not available | [4] |

| | | | | | | | | |
|---|---|---|---|---|---|---|---|---|
| Nygaard 2015 | 7 males and 2 females; Mean age: 32 ± 9 years; Baseline fitness levels: trained | RT | High: (-) | Single session; 60min | 15 min prior to exercise, immediately post-exercise, and thereafter 1, 2, 4, 6 and 24 hours post-exercise. | ~23% increase (at 1 hour post-exercise) | Baseline: 355 ± 50 ng/ml, post-exercise (at 1 hour): 437 ± 56 ng/ml | [14] |
| **Chronic exercise** | | | | | | | | |
| Adilakshmi 2023 | 50 males; aged 21 to 35 years. Baseline fitness levels: untrained | RT | High: (70% HRmax) | Five times per week; 8 weeks | Baseline and 30 min after the last intervention. | ~248% increase | Baseline: 48.05 ± 6.72 ng/ml, post-exercise: 167.39 ± 11.2 ng/ml | [15] |
| Scharhag-Rosenberger 2014 | 17 males and 20 females; Mean age: 47 ± 7 years; Baseline fitness levels: untrained | RT | Moderate: (64%-71% 1RM) | Three days per week; 24 weeks | After a 10 min supine resting period. | No significant change | Baseline: 314.6 ± 51.1 ng/ml, post-exercise: 337.0 ± 102.1 ng/ml | [16] |

Abbreviations: HICE: High-Intensity Continuous Exercise; HIIT: High-Intensity Interval Training; LICE: Low-Intensity Continuous Exercise; MICE: Moderate-Intensity Continuous Exercise; RT: Resistance Training; "(–)" indicates that the specific numerical threshold for exercise intensity was not reported in the original study; "~" indicates approximate percentage changes reported in the original studies.

**Supplementary Table S6.** Included studies investigating the effects of exercise intensity on circulating Fstl1 levels.

| Study ID | Study Population | Exercise Type | Exercise Intensity | Frequency & Duration | Time of Blood Sample | Changes in Concentration (percentage change) | Changes in Concentration (absolute values) | Reference |
|---|---|---|---|---|---|---|---|---|
| **Acute exercise** | | | | | | | | |
| Görgens 2013 | 8 males; Mean age: (-); Baseline fitness levels: trained | HICE | High: (70% $VO_2$max) | Single session; 60min | Baseline and immediately after the exercise session as well as 30 min and 120 min post-exercise. | ~29% increase (at 30 min post-exercise) | Baseline: 16.9 ± 3.5 ng/ml, post-exercise (at 30 min): 21.9 ± 2.9 ng/ml | [17] |
| Nam 2024 | 29 males; Mean age: 24.28 ± 3.53 years; Baseline fitness levels: untrained | HICE | High: (70% HRmax) | Single session; 60min | Baseline and immediately after the exercise session as well as 30 min, 60 min and 120 min post-exercise. | ~71% increase (immediately after exercise) | Baseline: 4.50 ± 1.25 ng/ml, post-exercise (immediately): 7.70 ± 2.76 ng/ml | [18] |
| Xu 2020 | 10 males and 11 females; Mean age: 25.2 ± 1.8 years; Baseline fitness levels: untrained | MICE | Moderate: (60% $VO_2$max) | Single session; 45min | Baseline and 45, 60, and 120 min following the 45 min bout of exercise. | ~19% increase (immediately after exercise) | Baseline: 6.68 ± 0.59 µg/L, post-exercise (immediately): 7.92 ± 0.72 µg/L | [19] |
| Kon 2021 | 8 males; Mean age: 20.3 ± 0.6 years; Baseline fitness levels: untrained | SIT | High: (maximal efforts) | Single session; Four bouts of 30-second all-out sprints | Baseline and immediately after the exercise session as well as 15, 30 and 120 min post-exercise. | ~73% increase (immediately after exercise) | Baseline: 151.87 ± 27.08 ng/ml, post-exercise (immediately): 261.87 ± 33.33 ng/ml | [20] |
| Ji 2024 | 9 males; Mean age: 24.0 ± 0.4 years; Baseline fitness levels: untrained | HIIT; VICE; MICE | MICE: (55% HRR); VICE: (85% HRR); HIIT: (all out) | MICE, VICE: Single session; 30min; HIIT: four bouts of 30s exercise with 4-minute rest intervals | Baseline and immediately after the exercise session as well as 30, 90 min post-exercise. | HIIT: ~175% increase (immediately after exercise); VICE: ~150% increase (at 30 min post-exercise); MICE: no significant change | HIIT: baseline: 1.8 ± 3.0 ng/ml, post-exercise (immediately): not available; VICE: baseline: 1.7 ± 1.96 ng/ml, post-exercise: not available; MICE: baseline: 2.4 ± 1.71 ng/ml, post-exercise: not available | [5] |

Abbreviations: HICE: High-Intensity Continuous Exercise; HIIT: High-Intensity Interval Training; MICE: Moderate-Intensity Continuous Exercise; SIT: Sprint Interval Training; VICE: Vigorous-Intensity Continuous Exercise; "(–)" indicates that the specific numerical threshold for exercise intensity was not reported in the original study; "~" indicates approximate percentage changes reported in the original studies.

**Supplementary Table S7.** Included studies investigating the effects of exercise intensity on circulating MSTN levels.

| Study ID | Study Population | Exercise Type | Exercise Intensity | Frequency & Duration | Time of Blood Sample | Changes in Concentration (percentage change) | Changes in Concentration (absolute values) | Reference |
|---|---|---|---|---|---|---|---|---|
| **Acute exercise** | | | | | | | | |
| Willoughby 2022 | 10 males; Mean age: 23.2 ± 4.68 years; Baseline fitness levels: trained | RT | High: (80% 1RM); Low: (50% 1RM) | Single session; To volitional failure | Baseline and at 3 and 24 hours post-exercise. | Low: ~17% decrease (at 24 hours post-exercise); High: ~35.7% decrease (at 24 hours post-exercise) | Low: baseline: 113.17 ± 38.9 ng/mL, post-exercise (at 24 hours): 93.39 ± 46.48 ng/mL; High: baseline: 117.21 ± 53.5 ng/mL, post-exercise (at 24 hours): 75.31 ± 51.23 ng/mL | [6] |
| Kazemi 2016 | 12 males; Mean age: 22.1 ± 2.1 years; Baseline fitness levels: trained | RT | Low: (55% 1RM) | 15 repetitions per set; 3 sets | Baseline and 24 hours post-exercise. | ~3.5% decrease (at 24 hours post-exercise) | Baseline: 9.12 ± 0.36 ng/mL, post-exercise (at 24 hours): 8.8 ± 0.36 ng/mL | [21] |
| **Chronic exercise** | | | | | | | | |
| Ziyaiyan 2023 | 9 males; Mean age: 15.17 ± 0.37 years; Baseline fitness levels: trained | HICT | High: (80%-85% HRmax) | Three times per week; 8 weeks | 48 hours before training and 48 hours after the last training session. | ~19% decrease | Baseline: 83.90 ± 22.34 ng/L, post-exercise: 67.55 ± 15.16 ng/L | [22] |
| Micielska 2021 | 9 males and 23 females; Mean age: 61 ± 12 years; Baseline fitness levels: untrained | MICE; HIIT | MICE: (60%-70% HRmax); HIIT: (90% HRmax) | MICE: Three times per week, 12week; HIIT: Three times per week, 2week | Baseline and 24 hours after completing the training program. | MICE: ~16% decrease; HIIT: no change | MICE: baseline: 4.45 ± 1.88 ng/dL, post-exercise: 3.71 ± 1.52 ng/dL; HIIT: baseline: 4.95 ± 1.40 ng/dL, post-exercise: 4.99 ± 1.80 ng/dL | [12] |
| Shabkhiz 2021 | 12 males, aged 65 to 78 years; Baseline fitness levels: untrained | RT | Moderate: (70% 1RM) | Three times per week; 12 weeks | One week before the training and 48 hours after the last training. | ~17.1% decrease | Baseline: 62.94 ± 18.36 ng/mL, post-exercise: 52.16 ± 13.91 ng/mL | [23] |

| | | | | | | | | |
|---|---|---|---|---|---|---|---|---|
| Arazi 2020 | 12 females; Mean age: 23.58 ± 3.84 years; Baseline fitness levels: untrained | RT | Moderate: (65%-80% 1RM) | Three times per week; 8 weeks | 48 hours before training and 48 hours after the last training. | ~23.2% decrease | Baseline: 632.46 ± 47.75 ng/L, post-exercise: 485.73 ± 67.49 ng/L | [24] |

Abbreviations: HICT: High-Intensity Circuit Training; HIIT: High-Intensity Interval Training; MICE: Moderate-Intensity Continuous Exercise; RT: Resistance Training; "(–)" indicates that the specific numerical threshold for exercise intensity was not reported in the original study; "~" indicates approximate percentage changes reported in the original studies.

**Supplementary Table S8.** Included studies investigating the effects of exercise intensity on circulating FGF21 levels.

| Study ID | Study Population | Exercise Type | Exercise Intensity | Frequency & Duration | Time of Blood Sample | Changes in Concentration (percentage change) | Changes in Concentration (absolute values) | Reference |
|---|---|---|---|---|---|---|---|---|
| **Acute Exercise** | | | | | | | | |
| Ji 2024 | 9 males; Mean age: 24.0 ± 0.4 years; Baseline fitness levels: untrained | HIIT; VICE; MICE | MICE: (55% HRR); VICE: (85% HRR); HIIT: (all out) | MICE, VICE: Single session, 30min; HIIT: four bouts of 30-second exercise with 4-minute rest intervals | Baseline and immediately after the exercise session as well as 30, 90 min post-exercise. | HIIT: ~75% increase (at 30 min post-exercise); VICE: ~40% increase (at 30 min post-exercise); MICE: no significant change | HIIT: baseline: 67.9 ± 49.8 pg/ml, post-exercise (immediately): not available; VICE: baseline: 70.9 ± 29.1 pg/ml, post-exercise: not available; MICE: baseline: 96.4 ± 128.1 pg/ml, post-exercise: not available | [5] |
| Morville 2018 | 10 males; Mean age: 24 ± 1 years; Baseline fitness levels: trained | HICE; RT | HICE: (70% $VO_2$peak); RT: High (90%-95% 10RM) | Single session; HICE: 60min; RT: 58-59min | Baseline and after the exercise at 15, 30, 60, 90, 120 and 180 min. | HICE: ~261% increase (at 60 min post-exercise); RT: No significant change | HICE: baseline: 46 ± 34.79 pg/mL, post-exercise (at 60 min): 166 ± 101.19 pg/mL; RT: baseline: 62 ± 53.76 pg/mL, post-exercise: not available | [7] |
| Wills 2019 | 10 males; Mean age: 26 ± 2 years; Baseline fitness levels: untrained | HICE; MICE | HICE: (75% $VO_2$peak); MICE: (55% $VO_2$peak) | Single session; HICE: 42 ± 6min; MICE: 42 ± 6min | Baseline and at 0, 1, 2, 4, and 7 hours post-exercise. | HICE: ~225% increase (at 1 hour post-exercise); MICE: ~13% increase (at 1hour post-exercise); | HICE: baseline: 65.65 ± 23.80 pg/ml, post-exercise (at 1 hour): 213.36 ± 31.18 pg/ml; MICE: baseline: 73.08 ± 17.86 pg/ml, post-exercise (at 1 hour): 82.58 ± 15.97 pg/ml | [8] |
| He 2019 | 14 males; Mean age: 23 ± 1 years; Baseline fitness levels: untrained | HICE; MICE | HICE: (85±8% $VO_2$max); MICE: (52±14% $VO_2$max) | Single session; HICE: 45min; MICE: 45min | Baseline, immediately after the training, and 1, 3, 24, 48, and 72 hours after each session. | HICE: ~194% increase (at 1 hour post-exercise); MICE: ~55% increase (at 24 hours post-exercise) | HICE: baseline: not available, post-exercise: not available; MICE: baseline: not available, post-exercise: not available | [9] |

| | | | | | | | | |
|---|---|---|---|---|---|---|---|---|
| He<br>2018 | 17 males;<br>Mean age:<br>23 ± 3 years;<br>Baseline fitness levels:<br>untrained | RT | Moderate:<br>(70-75% 1RM) | Single session; 50min | Baseline and 0, 1, 3,<br>24, 48, and 72 hours<br>post-exercise. | ~120% increase<br>(at 24 hours post-exercise) | Baseline: not available,<br>post-exercise: not available | [25] |
| **Chronic exercise** | | | | | | | | |
| Saeidi<br>2019 | 12 females;<br>Mean age:<br>58 ± 5 years;<br>Baseline fitness levels:<br>untrained | RT | Low:<br>(55% 1RM) | Three times per week;<br>8weeks | Baseline and post-training. | ~10.8% increase | Baseline: 254.2± 5.6 pg/ml,<br>post-exercise: 281.6± 5.5 pg/ml | [26] |

Abbreviations: HICE: High-Intensity Continuous Exercise; HIIT: High-Intensity Interval Training; MICE: Moderate-Intensity Continuous Exercise; RT: Resistance Training; VICE: Vigorous-Intensity Continuous Exercise; "~" indicates approximate percentage changes reported in the original studies.

**Supplementary Table S9.** Included studies investigating the effects of exercise intensity on circulating FST levels.

| Study ID | Study Population | Exercise Type | Exercise Intensity | Frequency & Duration | Time of Blood Sample | Changes in Concentration (percentage change) | Changes in Concentration (absolute values) | Reference |
|---|---|---|---|---|---|---|---|---|
| **Acute Exercise** | | | | | | | | |
| He 2019 | 14 males; Mean age: 23 ± 1 years; Baseline fitness levels: untrained | HICE; MICE | HICE: (85 ± 8% $VO_2$max); MICE: (52 ± 14% $VO_2$max) | Single session; HICE: 45min; HICE: 45min | Baseline, immediately after the training, and 1, 3, 24, 48, and 72 hours after each session. | HICE: ~259% increase (at 3 hours post-exercise); MICE: ~141% increase (at 3 hours post-exercise) | HICE: baseline: not available, post-exercise: not available; MICE: baseline: not available, post-exercise: not available | [9] |
| Wills 2019 | 10 males; Mean age: 26 ± 2 years; Baseline fitness levels: untrained | HICE; MICE | HICE: (75% $VO_2$peak); MICE: (55% $VO_2$peak) | Single session; HICE: 42 ± 6min; MICE: 42 ± 6min | Baseline and at 0, 1, 2, 4, and 7 hours post-exercise. | HICE: ~43% increase (at 4 hours post-exercise); MICE: ~30% increase (at 2 hours post-exercise); | HICE: baseline: 1392.07± 102.34 pg/ml, post-exercise (at 4 hours): 1990.66 ± 207.23 pg/ml; MICE: baseline: 1216.63 ± 277.78 pg/ml, post-exercise (at 2 hours): 1581.62 ± 219.39 pg/ml | [8] |
| He 2018 | 17 males; Mean age: 23 ± 3 years; Baseline fitness levels: untrained | RT | Moderate: (70-75% 1RM) | Single session; 50min | Baseline and 0, 1, 3, 24, 48, and 72 hours post-exercise. | ~90% increase (at 24 hours post-exercise) | Baseline: not available, post-exercise: not available | [25] |
| Willoughby 2022 | 10 males; Mean age: 23.2 ± 4.68 years; Baseline fitness levels: trained | RT | High: (80% 1RM); Low: (50% 1RM) | Single session; To volitional failure | Baseline and at 3 and 24 hours post-exercise. | Low: ~34.8% increase (at 24 hours post-exercise); High: ~22.8% increase (at 24 hours post-exercise) | Low: baseline: 1468 ± 354.92 pg/mL, post-exercise (at 24 hours): 1978.22 ± 531.06 pg/mL; High: baseline: 1523 ± 367.02 pg/mL, post-exercise (at 24 hours): 1869.78 ± 476.18 pg/mL | [6] |
| **Chronic exercise** | | | | | | | | |

| | | | | | | | | |
|---|---|---|---|---|---|---|---|---|
| Arazi 2020 | 12 females; Mean age: 23.58 ± 3.84 years; Baseline fitness levels: untrained | RT | Moderate: (65%-80% 1RM) | Three times per week; 8weeks | 48 hours before training and 48 hours after the last training. | ~49.15% increase | Baseline: 55.97 ± 6.72 ng/mL, post-exercise: 83.47 ± 7.84 ng/mL | [24] |

Abbreviations: HICE: High-Intensity Continuous Exercise; MICE: Moderate-Intensity Continuous Exercise; RT: Resistance Training; "~" indicates approximate percentage changes reported in the original studies.

**Supplementary Table S10.** Included studies investigating the effects of exercise intensity on circulating Leptin levels.

| Study ID | Study Population | Exercise Type | Exercise Intensity | Frequency & Duration | Time of Blood Sample | Changes in Concentration (percentage change) | Changes in Concentration (absolute values) | Reference |
|---|---|---|---|---|---|---|---|---|
| | **Acute exercise** | | | | | | | |
| Lakhdar 2013 | 8 males; Mean age: 20.7 ± 4.8 years; Baseline fitness levels: trained | HICE | High: (-) | Maximal Exercise, Single session; | Baseline, at the end and after 30, 60 min of recovery. | ~21.4% increase (immediately after exercise) | Baseline: 8.26 ± 4.85 μg/mL, post-exercise (immediately): 6.49 ± 4.59 μg/mL | [27] |
| | **Chronic exercise** | | | | | | | |
| Middelbeek 2021 | 22 males; Mean age: 48 ± 5 years; Baseline fitness levels: untrained | SIT; MICE | SIT: High: (all out); MICE: (60% $VO_2$peak) | SIT: six times per two weeks; MICE: six times per two weeks | Baseline and 48 h after the last exercise session. | SIT: ~23.7% decrease; MICE: ~11.1% decrease | SIT: baseline: 4814.6 ± 2305.27 ng/mL, post-exercise: 3673.5 ± 2305.27 ng/mL; MICE: baseline: 5079.9 ± 2242.94 ng/mL, post-exercise: 4516.2 ± 2242.94 ng/mL | [13] |
| Lakhdar 2013 | 8 males; Mean age: 20.7 ± 4.8 years; Baseline fitness levels: trained | HICE | High: (-) | Intense cycling training, six months | Baseline, at the end and after 30, 60 min of recovery. | ~44.9% decrease | Baseline: 8.26 ± 4.85 μg/mL, post-exercise: 4.55 ± 2.54 μg/mL | [27] |
| Zhang 2024 | 14 females; Mean age: 22.1 ± 1.6 years; Baseline fitness levels: untrained | HICT | High: (80%-82% HRmax) | Three times per week; 8 weeks | Baseline and post-training. | ~27.6% decrease | Baseline: 8.95 ± 5.00 ng/mL, post-training: 6.48 ± 4.88 ng/mL | [28] |
| Zaman 2021 | 20 males; aged 35 to 60 years; Baseline fitness levels: untrained | RT | Moderate to low: (50%-70% 1RM) | Three times per week; 12 weeks | Baseline, the sixth week and the twelfth week. | No significant change | Baseline: 12.10 ± 0.70 ng/mL, the sixth week: 11.85 ± 0.97 ng/mL, the twelfth week: 11.18 ± 0.89 ng/mL | [29] |

| | | | | | | | | |
|---|---|---|---|---|---|---|---|---|
| Saeidi 2019 | 12 females; Mean age: 58 ± 5 years; Baseline fitness levels: untrained | RT | Low: (55% 1RM) | Three times per week; 8 weeks | Baseline and post-training. | ~7.8% decrease | Baseline: 20.6 ± 0.4 ng/mL, post-training: 19 ± 0.49 ng/mL | [26] |

Abbreviations: HICE: High-Intensity Continuous Exercise; HICT: High-Intensity Circuit Training; MICE: Moderate-Intensity Continuous Exercise; RT: Resistance Training; SIT: Sprint Interval Training; "(–)" indicates that the specific numerical threshold for exercise intensity was not reported in the original study; "~" indicates approximate percentage changes reported in the original studies.

**Supplementary Table S11.** Included studies investigating the effects of exercise intensity on circulating Adiponectin levels.

| Study ID | Study Population | Exercise Type | Exercise Intensity | Frequency & Duration | Time of Blood Sample | Changes in Concentration (percentage change) | Changes in Concentration (absolute values) | Reference |
|---|---|---|---|---|---|---|---|---|
| | **Acute exercise** | | | | | | | |
| Mallardo 2024 | 15 males; Mean age: 25.3 ± 4.1 years; Baseline fitness levels: trained | HICE | High: (exhaustive exercise) | Single session; To exhaustive | Baseline, at 15 min and 24 hours post-exercise. | ~18.4% increase (at 24 hours post-exercise) | Baseline: 15.03 ± 2.2 μg/mL, post-exercise (at 24 hours): 17.8 ± 2.1 μg/mL | [30] |
| Kraemer 2003 | 6 males; Mean age: 23 ± 1.34 years; Baseline fitness levels: untrained | HICE | High: (79% $VO_2max$) | Single session; 30min | Baseline and immediately after the exercise session as well as 30 min post-exercise. | ~10% increase (immediately post-exercise) | Baseline: 7.45 ± 1.10 μg/mL, post-exercise (immediately): 8.18 ± 1.20 μg/mL | [31] |
| Numao 2008 | 8 males; Mean age: 24.9 ± 1.8 years; Baseline fitness levels: untrained | MICE | Moderate: (50% $VO_2peak$) | Single session; 60min | Baseline, 20, 40, 60 minutes after the start of exercise, and 30 minutes post-exercise. | No significant change | Baseline: not available， post-exercise: not available | [32] |
| | **Chronic exercise** | | | | | | | |
| Shing 2013 | 5 males and 2 females; Mean age: 19 ± 1.2 years; Baseline fitness levels: trained | HIIT | High: (70% HRmax) | Twice per week; 4 weeks | Baseline and post-training. | ~10.7% increase | Baseline: 12.40 ± 3.60 μg/mL, post-training: 13.73 ± 3.47 μg/mL | [33] |
| Benedetta 2023 | 57 females; Mean age: 58.7 ± 4.9 years; Baseline fitness levels: untrained | MICE | Moderate: (3 MET-hours) | Up to 1 hour per day; 24 months | Baseline and post-exercise. | No significant change | Baseline: not available， post-exercise: not available | [34] |

| | | | | | | | | |
|---|---|---|---|---|---|---|---|---|
| Jürimäe 2007 | 12 males;<br>Mean age:<br>20.8 ± 3.0 years;<br>Baseline fitness levels: trained | RT | Low:<br>(50% 1RM) | 24 weeks | Every four weeks. | No significant change | Baseline: 25.4 ± 9.9 μg/mL,<br>post-training: 26.3 ± 6.3 μg/mL | [35] |

Abbreviations: HICE: High-Intensity Continuous Exercise; HIIT: High-Intensity Interval Training; MICE: Moderate-Intensity Continuous Exercise; RT: Resistance Training; "~" indicates approximate percentage changes reported in the original studies.

**Supplementary Table S12.** Included studies investigating the effects of exercise intensity on circulating Apelin levels.

| Study ID | Study Population | Exercise Type | Exercise Intensity | Frequency & Duration | Time of Blood Sample | Changes in Concentration (percentage change) | Changes in Concentration (absolute values) | Reference |
|---|---|---|---|---|---|---|---|---|
| **Acute exercise** | | | | | | | | |
| Kon 2021 | 8 males; Mean age: 20.3 ± 0.6 years; Baseline fitness levels: untrained | SIT | High: (maximal efforts) | Single session; Four bouts of 30-second all-out sprints | Baseline and immediately after the exercise session as well as 15, 30 and 120 min post-exercise. | ~31% increase (immediately post-exercise) | Baseline: 274.87 ± 27.01 pg/mL, post-exercise (immediately): 360.07 ± 70.4 pg/mL | [20] |
| Son 2019 | 8 males; Mean age: 37.38 ± 9.75 years; Baseline fitness levels: untrained | A single bout of exhaustive exercise. | High: (exhaustive exercise) | Single session; To exhaustive | Baseline and immediately after the exercise session as well as 15, 30 min post-exercise. | No significant change | Baseline: 0.34 ± 0.10 ng/mL, post-exercise (immediately): 0.37 ± 0.09 ng/mL | [36] |
| Waller 2019 | 7 males and 5 females; Mean age: 22.8 ± 2.9 years; Baseline fitness levels: untrained | HICE | High: (maximal $VO_2max$); (70%-75% $VO_3max$) | Single session; maximal VO2max: 7.21 ± 0.3 min; 70%-75% VO2max: 30min | Baseline and immediately after the exercise session as well as 1, 24 hours post-exercise. | No significant change | Baseline: not available, post-exercise: not available | [37] |
| Ligetvári 2023 | 58 males; Mean age: 22.9 ± 4.7 years; Baseline fitness levels: trained | HICE | High (maximal cardiorespiratory exercise) | Single session; 11.2 ± 1.5 min | Baseline, immediately after the exercise, and 30 min post-exercise. | Apelin-13: ~16% increase (immediately post-exercise); Apelin-36: ~149% increase (immediately post-exercise) | Apelin-13: baseline: 144 ± 72.4 pg/mL, post-exercise (immediately): 167 ± 71.5 pg/mL; Apelin-36: baseline: 60.2 ± 37.85 pg/mL, post-exercise (immediately): 150 ± 50.36 pg/mL | [38] |
| **Chronic exercise** | | | | | | | | |

| | | | | | | | | |
|---|---|---|---|---|---|---|---|---|
| Fujie 2022 | 7 males and 10 females; Mean age: 65.5 ± 2.0 years; Baseline fitness levels: untrained | HICE | High: (60%-70% $VO_2$peak) | Three times per week; 8 weeks | Every two weeks. | ~127% increase | Baseline: 2.33 ± 0.78 ng/mL, post-exercise: 5.28 ± 0.62 ng/mL | [39] |
| Fujie 2014 | 7 males and 11 females; Mean age: 66.4 ± 2.1 years; Baseline fitness levels: untrained | HICE | High: (60%-70% $VO_2$peak) | Three times per week; 8 weeks | Baseline and 48 hours after the last exercise session. | ~116% increase | Baseline: 2.31 ± 0.85 ng/mL, post-exercise: 5.00 ± 1.65 ng/mL | [40] |

Abbreviations: HICE: High-Intensity Continuous Exercise; SIT: Sprint Interval Training. "~" indicates approximate percentage changes reported in the original studies.

**Supplementary Table S13.** Included studies investigating the effects of exercise intensity on circulating BDNF levels.

| Study ID | Study Population | Exercise Type | Exercise Intensity | Frequency & Duration | Time of Blood Sample | Changes in Concentration (percentage change) | Changes in Concentration (absolute values) | Reference |
|---|---|---|---|---|---|---|---|---|
| **Acute exercise** | | | | | | | | |
| Reycraft 2019 | 8 males； Mean age: 23.1 ± 3.0 years; Baseline fitness levels: untrained | MICE; VICE; SIT | MICE: (65% $VO_2$max); VICE: (85% $VO_2$max); SIT, high: (maximal effort) | Single session; 30min | Baseline and immediately after the exercise session as well as 30 min and 90 min post-exercise. | Increased in an intensity-dependent manner, SIT induced the highest BDNF concentration, ~190% increase (immediately post-exercise) | SIT: baseline: 3168 ± 420 pg/mL, post-exercise: not available; VICE: baseline: 5470 ± 1535 pg/mL, post-exercise: not available; MICE: baseline: 4775 ± 1095 pg/mL, post-exercise: not available | [10] |
| Ji 2024 | 9 males; Mean age: 24.0 ± 0.4 years; Baseline fitness levels: untrained | HIIT; VICE; MICE | MICE (55% HRR); VICE (85% HRR); HIIT (all out) | MICE, VICE: Single session; 30min; HIIT: four bouts of 30-second exercise with 4-minute rest intervals | Baseline and immediately after the exercise session as well as 30 min and 90 min post-exercise. | HIIT: ~55% increase (immediately post-exercise); VICE: ~25% increase (immediately post-exercise); MICE: no significant change | HIIT: baseline: 20972.6 ± 6599.1 pg/ml, post-exercise (immediately): not available; VICE: baseline: 21141.9 ± 8030.1 pg/ml, post-exercise: not available; MICE: baseline: 25385.8 ± 9929.1 pg/ml, post-exercise: not available | [5] |
| Aktitiz 2022 | 20 males; Mean age: 29.1 ± 5.8 years; Baseline fitness levels: trained | HIIT | High: (100% $VO_2$peak) | Single session; 10×1min | Baseline and post-exercise. | ~236% increase (post-exercise) | Baseline: 1326 ± 424.4 pg/mL, post-exercise: 3828 ± 1122 pg/mL | [41] |
| Arazi 2021 | 20 males; Mean age: 60.8 ± 1.7 years; Baseline fitness levels: trained | RT; MICE | RT, moderate: (65%-70% 1RM); MICE: (65%-70% HRmax) | Single session; 45min | Baseline and post exercise at 10 min. | RT: ~16% increase (post-exercise at 10 min); MICE : ~13% increase (post-exercise at 10 min) | RT: baseline: 3.58 ± 1.05 ng/mL, post-exercise: 4.16 ± 1.82 ng/mL; MICE : baseline: 4.13 ± 3.65 ng/mL, post-exercise: 4.68 ± 3.9 ng/mL | [42] |

| | | | | | | | | |
|---|---|---|---|---|---|---|---|---|
| Borges 2024 | 14 males;<br>Mean age:<br>41.0 ± 5.8 years;<br>Baseline fitness levels: trained | RT | High:<br>(80% 1RM);<br>Moderate:<br>(60% 1RM) | Single session;<br>four groups | Baseline and immediately after the training session as well as 60 min post-exercise. | High:<br>~275% increase (post-exercise);<br>Moderate: no significant change | High: baseline: 461 pg/mL,<br>post-exercise: 1730 pg/mL,<br>(the standard deviation was not available)；<br>Moderate: not available | [11] |
| Lira 2020 | 12 young men;<br>Mean age:<br>25.3 ± 5.9 years;<br>Baseline fitness levels: trained | RT | Moderate:<br>(65% 1RM) | Single session;<br>five groups,<br>ten repetitions per group | Baseline, post-exercise immediately,<br>1 hour post-exercise,<br>and 2 hours post-exercise. | ~87% increase (immediately post-exercise) | Baseline: 6090.99 ± 2683.29 pg/ml,<br>post-exercise (immediately):<br>11390.15 ± 3743.75 pg/ml | [43] |
| **Chronic exercise** | | | | | | | | |
| Ji 2023 | 7 males and 13 females;<br>Mean age:<br>22.26 ± 1.68 years;<br>Baseline fitness levels: untrained | MICE | Moderate:<br>(55%-70% HRmax) | Five times per week;<br>4 weeks | One day before and after the exercise. | No significant change | Baseline: 4.63 ± 11.63 ng/mL,<br>post-training: 4.98 ± 10.96 ng/mL | [44] |
| Forti 2015 | 9 males;<br>Mean age:<br>68.05 ± 6.4 years;<br>Baseline fitness levels: untrained | RT | Low:<br>(20%-40% 1 RM) | Three times per week;<br>12 weeks | Baseline and after 12 weeks (24 hours-48 hours after the last training). | ~22.9% increase | Baseline: 34.9 ± 10.7 ng/mL,<br>post-training: 42.9 ± 11.9 ng/mL | [45] |

Abbreviations: HICE: High-Intensity Continuous Exercise; HIIT: High-Intensity Interval Training; MICE: Moderate-Intensity Continuous Exercise; RT: Resistance Training; SIT: Sprint Interval Training; VICE: Vigorous-Intensity Continuous Exercise; "~" indicates approximate percentage changes reported in the original studies.

## References


1. Colpitts, B.H.; Rioux, B.V.; Eadie, A.L.; Brunt, K.R.; Sénéchal, M. Irisin response to acute moderate intensity exercise and high intensity interval training in youth of different obesity statuses: A randomized crossover trial. *Physiol Rep* **2022**, *10*, e15198, doi:10.14814/phy2.15198.
2. Tsai, C.L.; Pan, C.Y.; Tseng, Y.T.; Chen, F.C.; Chang, Y.C.; Wang, T.C. Acute effects of high-intensity interval training and moderate-intensity continuous exercise on BDNF and irisin levels and neurocognitive performance in late middle-aged and older adults. *Behav Brain Res* **2021**, *413*, 113472, doi:10.1016/j.bbr.2021.113472.
3. Tsuchiya, Y.; Ando, D.; Goto, K.; Kiuchi, M.; Yamakita, M.; Koyama, K. High-intensity exercise causes greater irisin response compared with low-intensity exercise under similar energy consumption. *Tohoku J Exp Med* **2014**, *233*, 135-140, doi:10.1620/tjem.233.135.
4. Huh, J.Y.; Mougios, V.; Kabasakalis, A.; Fatouros, I.; Siopi, A.; Douroudos, II; Filippaios, A.; Panagiotou, G.; Park, K.H.; Mantzoros, C.S. Exercise-induced irisin secretion is independent of age or fitness level and increased irisin may directly modulate muscle metabolism through AMPK activation. *J Clin Endocrinol Metab* **2014**, *99*, E2154-2161, doi:10.1210/jc.2014-1437.
5. Ji, M.; Cho, C.; Lee, S. Acute effect of exercise intensity on circulating FGF-21, FSTL-1, cathepsin B, and BDNF in young men. *J Exerc Sci Fit* **2024**, *22*, 51-58, doi:10.1016/j.jesf.2023.11.002.
6. Willoughby, D.S.; Cardaci, T.D.; Machek, S.B.; Wilburn, D.T.; Heileson, J.L. Resistance Exercise-Induced Increases in Muscle Myostatin mRNA and Protein Expression Are Subsequently Decreased in Circulation in the Presence of Increased Levels of the Extracellular Matrix Stabilizing Protein Decorin. *Journal of sports science & medicine* **2022**, *21*, 616-624, doi:10.52082/jssm.2022.616.
7. Morville, T.; Sahl, R.E.; Trammell, S.A.; Svenningsen, J.S.; Gillum, M.P.; Helge, J.W.; Clemmensen, C. Divergent effects of resistance and endurance exercise on plasma bile acids, FGF19, and FGF21 in humans. *JCI Insight* **2018**, *3*, doi:10.1172/jci.insight.122737.
8. Willis, S.A.; Sargeant, J.A.; Thackray, A.E.; Yates, T.; Stensel, D.J.; Aithal, G.P.; King, J.A. Effect of exercise intensity on circulating hepatokine concentrations in healthy men. *Appl Physiol Nutr Metab* **2019**, *44*, 1065-1072, doi:10.1139/apnm-2018-0818.
9. He, Z.; Tian, Y.; Valenzuela, P.L.; Huang, C.; Zhao, J.; Hong, P.; He, Z.; Yin, S.; Lucia, A. Myokine/Adipokine Response to "Aerobic" Exercise: Is It Just a Matter of Exercise Load? *Frontiers in physiology* **2019**, *10*, 691, doi:10.3389/fphys.2019.00691.
10. Reycraft, J.T.; Islam, H.; Townsend, L.K.; Hayward, G.C.; Hazell, T.J.; Macpherson, R.E.K. Exercise Intensity and Recovery on Circulating Brain-derived Neurotrophic Factor. *Med Sci Sports Exerc* **2020**, *52*, 1210-1217, doi:10.1249/mss.0000000000002242.
11. Borges Junior, M.; Tavares, L.F.J.; Nagata, G.Y.; Barroso, L.S.S.; Fernandes, H.B.; Souza-Gomes, A.F.; Miranda, A.S.; Nunes-Silva, A. Impact of Strength Training Intensity on Brain-derived Neurotrophic Factor. *Int J Sports Med* **2024**, *45*, 155-161, doi:10.1055/a-2197-1201.
12. Micielska, K.; Flis, M.; Kortas, J.A.; Rodziewicz-Flis, E.; Antosiewicz, J.; Wochna, K.;

Lombardi, G.; Ziemann, E. Nordic Walking Rather Than High Intensity Interval Training Reduced Myostatin Concentration More Effectively in Elderly Subjects and the Range of This Drop Was Modified by Metabolites of Vitamin D. *Nutrients* **2021**, *13*, doi:10.3390/nu13124393.

13. Middelbeek, R.J.W.; Motiani, P.; Brandt, N.; Nigro, P.; Zheng, J.; Virtanen, K.A.; Kalliokoski, K.K.; Hannukainen, J.C.; Goodyear, L.J. Exercise intensity regulates cytokine and klotho responses in men. *Nutr Diabetes* **2021**, *11*, 5, doi:10.1038/s41387-020-00144-x.
14. Nygaard, H.; Slettaløkken, G.; Vegge, G.; Hollan, I.; Whist, J.E.; Strand, T.; Rønnestad, B.R.; Ellefsen, S. Irisin in blood increases transiently after single sessions of intense endurance exercise and heavy strength training. *PloS one* **2015**, *10*, e0121367, doi:10.1371/journal.pone.0121367.
15. Adilakshmi, P.; Suganthi, V.; Rao, K.S.; Mahendran, K.B. Effect of High-Intensity Resistance Training Versus Endurance Training on Irisin and Adipomyokine Levels in Healthy Individuals: An 8-Week Interventional Study. *Cureus* **2023**, *15*, e46483, doi:10.7759/cureus.46483.
16. Scharhag-Rosenberger, F.; Meyer, T.; Wegmann, M.; Ruppenthal, S.; Kaestner, L.; Morsch, A.; Hecksteden, A. Irisin does not mediate resistance training-induced alterations in resting metabolic rate. *Med Sci Sports Exerc* **2014**, *46*, 1736-1743, doi:10.1249/mss.0000000000000286.
17. Görgens, S.W.; Raschke, S.; Holven, K.B.; Jensen, J.; Eckardt, K.; Eckel, J. Regulation of follistatin-like protein 1 expression and secretion in primary human skeletal muscle cells. *Arch Physiol Biochem* **2013**, *119*, 75-80, doi:10.3109/13813455.2013.768270.
18. Nam, J.S.; Park, S.J.; Ahn, C.W.; Cho, E.S.; Kim, H.J.; Kim, Y. Follistatin-like 1 is a myokine regulating lipid mobilization during endurance exercise and recovery. *Obesity (Silver Spring)* **2024**, *32*, 352-362, doi:10.1002/oby.23949.
19. Xu, X.; Zhang, T.; Mokou, M.; Li, L.; Li, P.; Song, J.; Liu, H.; Zhu, Z.; Liu, D.; Yang, M.; et al. Follistatin-like 1 as a Novel Adipomyokine Related to Insulin Resistance and Physical Activity. *J Clin Endocrinol Metab* **2020**, *105*, doi:10.1210/clinem/dgaa629.
20. Kon, M.; Ebi, Y.; Nakagaki, K. Effects of acute sprint interval exercise on follistatin-like 1 and apelin secretions. *Arch Physiol Biochem* **2021**, *127*, 223-227, doi:10.1080/13813455.2019.1628067.
21. Kazemi, F. The correlation of resistance exercise-induced myostatin with insulin resistance and plasma cytokines in healthy young men. *J Endocrinol Invest* **2016**, *39*, 383-388, doi:10.1007/s40618-015-0373-9.
22. Ziyaiyan, A.; Kordi, M.; Hofmeister, M.; Chamari, K.; Moalla, W.; Gaeini, A.A. High-intensity circuit training change serum myostatin but not myogenin in adolescents' soccer players: a quasi-experimental study. *BMC Sports Sci Med Rehabil* **2023**, *15*, 15, doi:10.1186/s13102-023-00627-1.
23. Shabkhiz, F.; Khalafi, M.; Rosenkranz, S.; Karimi, P.; Moghadami, K. Resistance training attenuates circulating FGF-21 and myostatin and improves insulin resistance in elderly men with and without type 2 diabetes mellitus: A randomised controlled clinical trial. *Eur J Sport Sci* **2021**, *21*, 636-645, doi:10.1080/17461391.2020.1762755.
24. Arazi, H.; Salek, L.; Nikfal, E.; Izadi, M.; Tufano, J.J.; Elliott, B.T.; Brughelli, M.

Comparable endocrine and neuromuscular adaptations to variable vs. constant gravity-dependent resistance training among young women. *J Transl Med* **2020**, *18*, 239, doi:10.1186/s12967-020-02411-y.
25. He, Z.; Tian, Y.; Valenzuela, P.L.; Huang, C.; Zhao, J.; Hong, P.; He, Z.; Yin, S.; Lucia, A. Myokine Response to High-Intensity Interval vs. Resistance Exercise: An Individual Approach. *Frontiers in physiology* **2018**, *9*, 1735, doi:10.3389/fphys.2018.01735.
26. Saeidi, A.; Jabbour, G.; Ahmadian, M.; Abbassi-Daloii, A.; Malekian, F.; Hackney, A.C.; Saedmocheshi, S.; Basati, G.; Ben Abderrahman, A.; Zouhal, H. Independent and Combined Effects of Antioxidant Supplementation and Circuit Resistance Training on Selected Adipokines in Postmenopausal Women. *Frontiers in physiology* **2019**, *10*, 484, doi:10.3389/fphys.2019.00484.
27. Lakhdar, N.; Ben Saad, H.; Denguezli, M.; Zaouali, M.; Zbidi, A.; Tabka, Z.; Bouassida, A. Effects of intense cycling training on plasma leptin and adiponectin and its relation to insulin resistance. *Neuro Endocrinol Lett* **2013**, *34*, 229-235.
28. Zhang, B.; Zheng, C.; Hu, M.; Fang, Y.; Shi, Y.; Tse, A.C.; Lo, S.K.; Wong, S.H.; Sun, F. The effect of different high-intensity interval training protocols on cardiometabolic and inflammatory markers in sedentary young women: A randomized controlled trial. *J Sports Sci* **2024**, *42*, 751-762, doi:10.1080/02640414.2024.2363708.
29. Zaman, G.S.; Abohashrh, M.; Ahmad, I.; Dera, A.A.; Alshahrani, M.S.; Ahmad, I.; Alam, M.M.; Mahmood, S.E.; Mansuri, N.; Irfan, S.; et al. The Impact of Body Resistance Training Exercise on Biomedical Profile at High Altitude: A Randomized Controlled Trial. *Biomed Res Int* **2021**, *2021*, 6684167, doi:10.1155/2021/6684167.
30. Mallardo, M.; Tommasini, E.; Missaglia, S.; Pecci, C.; Rampinini, E.; Bosio, A.; Morelli, A.; Daniele, A.; Nigro, E.; Tavian, D. Effects of Exhaustive Exercise on Adiponectin and High-Molecular-Weight Oligomer Levels in Male Amateur Athletes. *Biomedicines* **2024**, *12*, doi:10.3390/biomedicines12081743.
31. Kraemer, R.R.; Aboudehen, K.S.; Carruth, A.K.; Durand, R.T.; Acevedo, E.O.; Hebert, E.P.; Johnson, L.G.; Castracane, V.D. Adiponectin responses to continuous and progressively intense intermittent exercise. *Med Sci Sports Exerc* **2003**, *35*, 1320-1325, doi:10.1249/01.Mss.0000079072.23998.F3.
32. Numao, S.; Suzuki, M.; Matsuo, T.; Nomata, Y.; Nakata, Y.; Tanaka, K. Effects of acute aerobic exercise on high-molecular-weight adiponectin. *Med Sci Sports Exerc* **2008**, *40*, 1271-1276, doi:10.1249/MSS.0b013e31816a9ee5.
33. Shing, C.M.; Webb, J.J.; Driller, M.W.; Williams, A.D.; Fell, J.W. Circulating adiponectin concentration and body composition are altered in response to high-intensity interval training. *J Strength Cond Res* **2013**, *27*, 2213-2218, doi:10.1519/JSC.0b013e31827e1644.
34. Bendinelli, B.; Masala, G.; Bella, C.D.; Assedi, M.; Benagiano, M.; Pratesi, S.; Ermini, I.; Occhini, D.; Castaldo, M.; Saieva, C.; et al. Adipocytokine plasma level changes in a 24-month dietary and physical activity randomised intervention trial in postmenopausal women. *Eur J Nutr* **2023**, *62*, 1185-1194, doi:10.1007/s00394-022-03055-y.
35. Jürimäe, J.; Purge, P.; Jürimäe, T. Effect of prolonged training period on plasma adiponectin in elite male rowers. *Horm Metab Res* **2007**, *39*, 519-523, doi:10.1055/s-2007-984397.
36. Son, J.S.; Chae, S.A.; Park, B.I.; Du, M.; Song, W. Plasma apelin levels in

overweight/obese adults following a single bout of exhaustive exercise: A preliminary cross-sectional study. *Endocrinologia, diabetes y nutricion* **2019**, *66*, 278-290, doi:10.1016/j.endinu.2018.12.005.

37. Waller, J.D.; McNeill, E.H.; Zhong, F.; Vervaecke, L.S.; Goldfarb, A.H. Plasma Apelin Unchanged With Acute Exercise Insulin Sensitization. *Journal of sports science & medicine* **2019**, *18*, 537-543.
38. Ligetvári, R.; Szokodi, I.; Far, G.; Csöndör, É.; Móra, Á.; Komka, Z.; Tóth, M.; Oláh, A.; Ács, P. Apelin as a Potential Regulator of Peak Athletic Performance. *International journal of molecular sciences* **2023**, *24*, doi:10.3390/ijms24098195.
39. Fujie, S.; Sanada, K.; Hamaoka, T.; Iemitsu, M. Time-dependent relationships between exercise training-induced changes in nitric oxide production and hormone regulation. *Experimental gerontology* **2022**, *166*, 111888, doi:10.1016/j.exger.2022.111888.
40. Fujie, S.; Sato, K.; Miyamoto-Mikami, E.; Hasegawa, N.; Fujita, S.; Sanada, K.; Hamaoka, T.; Iemitsu, M. Reduction of arterial stiffness by exercise training is associated with increasing plasma apelin level in middle-aged and older adults. *PloS one* **2014**, *9*, e93545, doi:10.1371/journal.pone.0093545.
41. Aktitiz, S.; Atakan, M.M.; Turnagöl, H.H.; Koşar Ş, N. Interleukin-6, undercarboxylated osteocalcin, and brain-derived neurotrophic factor responses to single and repeated sessions of high-intensity interval exercise. *Peptides* **2022**, *157*, 170864, doi:10.1016/j.peptides.2022.170864.
42. Arazi, H.; Babaei, P.; Moghimi, M.; Asadi, A. Acute effects of strength and endurance exercise on serum BDNF and IGF-1 levels in older men. *BMC Geriatr* **2021**, *21*, 50, doi:10.1186/s12877-020-01937-6.
43. Lira, F.S.; Conrado de Freitas, M.; Gerosa-Neto, J.; Cholewa, J.M.; Rossi, F.E. Comparison Between Full-Body vs. Split-Body Resistance Exercise on the Brain-Derived Neurotrophic Factor and Immunometabolic Response. *J Strength Cond Res* **2020**, *34*, 3094-3102, doi:10.1519/jsc.0000000000002653.
44. Ji, Y.; Ni, X.; Zheng, K.; Jiang, Y.; Ren, C.; Zhu, H.; Xiao, M.; Wang, T. Synergistic effects of aerobic exercise and transcranial direct current stimulation on executive function and biomarkers in healthy young adults. *Brain Res Bull* **2023**, *202*, 110747, doi:10.1016/j.brainresbull.2023.110747.
45. Forti, L.N.; Van Roie, E.; Njemini, R.; Coudyzer, W.; Beyer, I.; Delecluse, C.; Bautmans, I. Dose-and gender-specific effects of resistance training on circulating levels of brain derived neurotrophic factor (BDNF) in community-dwelling older adults. *Experimental gerontology* **2015**, *70*, 144-149, doi:10.1016/j.exger.2015.08.004.
